# Zinc oxide nanoparticles exacerbate skin epithelial cell damage by upregulating pro-inflammatory cytokines and exosome secretion in M1 macrophages following UVB irradiation-induced skin injury

**DOI:** 10.1186/s12989-024-00571-z

**Published:** 2024-02-28

**Authors:** Bour-Jr Wang, Yu-Ying Chen, Hui-Hsuan Chang, Rong-Jane Chen, Ying-Jan Wang, Yu-Hsuan Lee

**Affiliations:** 1 Department of Cosmetic Science and Institute of Cosmetic Science, Chia Nan University of Pharmacy and Science, Tainan, 71710 Taiwan; 2 Department of Occupational and Environmental Medicine, National Cheng Kung University Hospital, Tainan, 70403 Taiwan; 3 Department of Environmental and Occupational Health, College of Medicine, National Cheng Kung University, 138 Sheng-Li Road, Tainan, 70428 Taiwan; 4 Department of Food Safety/Hygiene and Risk Management, College of Medicine, National Cheng Kung University, 138 Sheng-Li Road, Tainan, 70428 Taiwan; 5 Department of Medical Research, China Medical University Hospital, China Medical University, Taichung, 406040 Taiwan; 6 Department of Cosmeceutics, China Medical University, Taichung, 406040 Taiwan

**Keywords:** Zinc oxide nanoparticle, Transepidermal water loss, Lysosomal impairment, Autophagy dysfunction, Macrophage polarization, Exosome

## Abstract

**Background:**

Zinc oxide nanoparticles (ZnONPs) are common materials used in skin-related cosmetics and sunscreen products due to their whitening and strong UV light absorption properties. Although the protective effects of ZnONPs against UV light in intact skin have been well demonstrated, the effects of using ZnONPs on damaged or sunburned skin are still unclear. In this study, we aimed to reveal the detailed underlying mechanisms related to keratinocytes and macrophages exposed to UVB and ZnONPs.

**Results:**

We demonstrated that ZnONPs exacerbated mouse skin damage after UVB exposure, followed by increased transepidermal water loss (TEWL) levels, cell death and epithelial thickness. In addition, ZnONPs could penetrate through the damaged epithelium, gain access to the dermis cells, and lead to severe inflammation by activation of M1 macrophage. Mechanistic studies indicated that co-exposure of keratinocytes to UVB and ZnONPs lysosomal impairment and autophagy dysfunction, which increased cell exosome release. However, these exosomes could be taken up by macrophages, which accelerated M1 macrophage polarization. Furthermore, ZnONPs also induced a lasting inflammatory response in M1 macrophages and affected epithelial cell repair by regulating the autophagy-mediated NLRP3 inflammasome and macrophage exosome secretion.

**Conclusions:**

Our findings propose a new concept for ZnONP-induced skin toxicity mechanisms and the safety issue of ZnONPs application on vulnerable skin. The process involved an interplay of lysosomal impairment, autophagy-mediated NLRP3 inflammasome and macrophage exosome secretion. The current finding is valuable for evaluating the effects of ZnONPs for cosmetics applications.

**Supplementary Information:**

The online version contains supplementary material available at 10.1186/s12989-024-00571-z.

## Introduction

Currently, nanomaterials are becoming extremely popular in many industry fields and are applied in several basic commodities on the market due to their unique physical and chemical properties [1, 2]. Among them, zinc oxide nanoparticles (ZnONPs) are one of the common materials to add to skin-related cosmetics and sunscreen products. Because of their low irritation, hyposensitivity, high dispersion, transparency, and antibacterial properties, ZnONPs are indispensable additives in skin products [3, 4]. Sunburn skin normally has a vulnerable skin barrier that alters skin functions, increases skin water loss, and causes skin inflammation, aging, and hyperpigmentation [5, 6]. Dermatologists recommend applying sunscreen to prevent sunburn, and the typical dose of ZnONPs in sunscreen is approximately 2 mg/cm^2^ [7, 8]. However, further verification is required to determine whether the utilization of the new formulation of ZnONPs as a sunscreen ingredient will elevate the burden on skin tissue, particularly on damaged or sunburned skin, and whether it will heighten the potential for ZnONPs to enter human tissues. While studies have indicated that ZnONPs cannot penetrate into the deeper layers of intact skin [9, 10], exposure of quantum dots on sunburned skin has demonstrated their penetration into the dermal layers [11]. Therefore, it is imperative to conduct additional investigations to ascertain whether ZnONPs persist within the superficial stratum of the skin or possess the capability to permeate into the deeper layers of compromised skin, thereby potentially intensifying skin damage.

UVB (Ultraviolet B) exposure has been shown to induce epidermal cells to secrete molecules, which in turn induce the release of inflammatory mediators from the dermis, as well as attract inflammatory cells, such as monocytes or macrophages, from circulation into specific regions of the skin [12]. As an essential component of innate immunity, macrophages can respond to various intrinsic or environmental stresses and acquire distinct functional phenotypes by undergoing different phenotypic polarizations [13]. Two major macrophage polarization phenotypes have been classified: activated macrophages or M1 (proinflammatory) and alternatively activated macrophages or M2 (anti-inflammatory), each of which regulates specific immune responses [14]. M1 macrophages produce many proinflammatory cytokines, including TNF-α, IL-1, and IL-6 [15]. The increased M1/M2 ratio has been suggested to be related to the development of inflammatory bowel disease and osteoarthritis, as well as hindering wound healing [16–19]. Therefore, the transition between M1 and M2 macrophages in skin tissue may determine the repair of damaged skin.

Protein degradation and renewal of cellular organelles are necessary for cell survival. If this process is disrupted, it may cause abnormal cell growth and cell death, causing various diseases [20]. Autophagy is a process of cell self-protection that plays an important role in maintaining eukaryotic cell homeostasis and removing damaged organelles. By transmitting cytoplasmic contents to lysosomes for degradation, autophagy balances cell composition synthesis, decomposition and reuse. The autophagy machinery constitutes a key cellular monitoring system that prevents excessive inflammation, including inhibition of the NLRP3 inflammasome, which can be regarded as an anti-inflammatory defense mechanism [21, 22]. Hence, defects in autophagic processes could be one of the vital cellular mechanisms for inducing inflammatory skin diseases [23]. Our previous studies have described that silver nanoparticles have the ability to induce endoplasmic reticulum (ER) stress and autophagy dysfunction in fibroblasts [24]. Recently, autophagy-lysosomal pathways, as an important mechanism for regulating many diseases, have been proven to be closely related to the release of extracellular vesicles (EVs) in organisms [25].

EVs are nanoscale extracellular vesicles derived from the endocytic pathway. It is mainly composed of a double-layer phospholipid membrane, and most of the contents are proteins, growth factors, cytokines, lipids, miRNA, mRNA, and DNA, which can serve as mediators to regulate cell-to-cell communications [26]. In the past decade, researchers have paid more attention to the role of EVs in physiology and disease, especially exosomes [27]. The smallest EV types are exosomes, which have been implicated in reducing inflammation for anti-obesity effects [28], increasing proangiogenic factors [29] and promoting tumor progression [30–32] by polarizing M2 macrophages. Interestingly, polarizing M1 macrophages were significantly increased when macrophages were treated with exosomes derived from HaCaT cells in our present study. Recently, we demonstrated that HaCaT cells exposed to UVB combined with ZnONPs can increase the secretion of exosomes, which contain NLRP3 inflammasome complex proteins [33]. However, it is still unclear whether the proteins contained in exosomes can induce further inflammatory responses in M1 macrophages. In this study, we propose a new concept in which ZnONPs enhance the alteration of the macrophage phenotype and increase exosome secretion to interfere with the mechanisms of skin inflammation after UVB exposure in keratinocytes.

## Results

### NH_2_-ZnONP (ZnONP) and R6G-ZnONPs properties and experimental design for applying ZnONPs to mouse skin

**Fig. 1:**
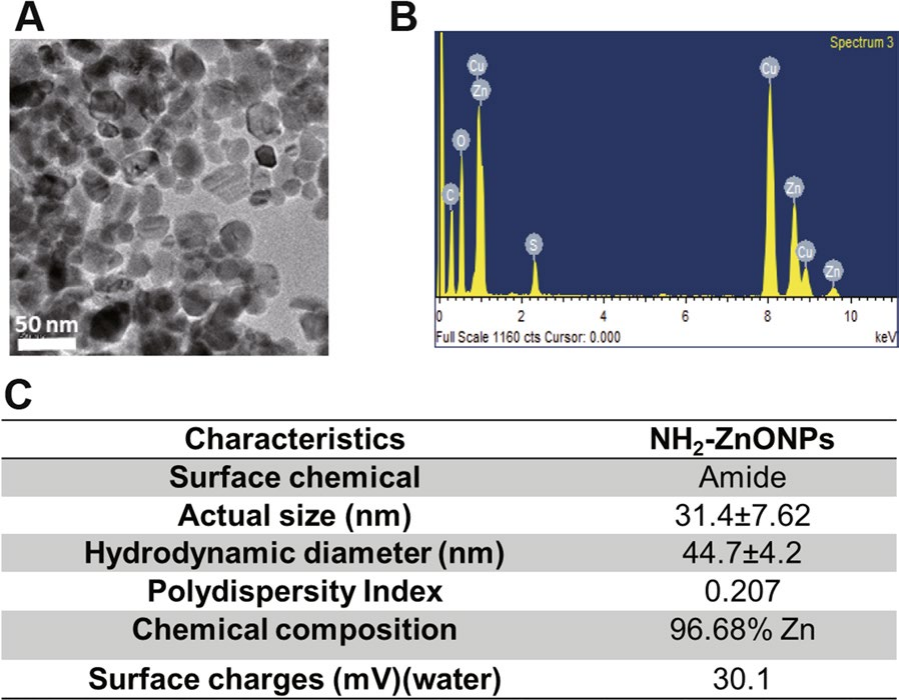
Characterization of ZnONPs. **A** Transmission electron microscopy (TEM) images of ZnONPs. **B** The composition of ZnONPs was analyzed by energy-dispersive X-ray (EDX) spectrometry. **C** Summary of the physicochemical properties of ZnONPs

In our study, we chose APTES (3-aminopropyl) tiethoxysilane as an amino-silane, which is mainly used as a dispersant to synthesize NH_2_-ZnONPs (ZnONPs). Then, we analyzed several physical and chemical properties of it (Fig. 1). The TEM (Transmission electron microscope) image showed that the appearance of ZnONPs was mostly spherical (Fig. 1A), and the chemical composition of ZnONPs showed that after excluding the carbon-coated copper mesh, the zinc content reached more than 90%, and there were no other significant impurities (Fig. 1B). We unified the measured values of various physical and chemical properties in Fig. 1C. The actual particle size of ZnONPs was 31.4 ± 7.62 nm. Compared with the actual particle size, the hydrodynamic diameter was increased to 44.7 ± 4.2 nm. The polydispersity index (PI) of ZnONPs is 0.207, indicating that it had good dispersion without significant agglomeration. We also analyzed the charges of ZnONPs. Due to amine modification, ZnONPs presented a positive charge, and the surface charge was 30.1 mV, which could prevent ZnONPs from aggregating and maintain their stability. Fluorescence emission spectrum of ZnONPs indicated a broad emission region around 500–600 nm were shown in the spectrum, which indicates a green-yellow fluorescence emission of ZnONPs were observed (Additional file 1: Fig. S1). To study NP distribution in skin, we conjugated the fluorescent dye rhodamine 6G (R6G) to ZnONPs. The chemical bonding and stability of R6G and ZnONPs was analyzed using X-ray photoelectron spectroscopy (XPS) (Additional file 1: Fig.S2 A). The XPS data indicated that R6G formed new C–O bonds on the ZnONP surface from the original Zn–O metal oxide bonding (Additional file 1: Fig. S2 B–D). The C–O bonding ratio showed no difference after 72 h of incubation, indicating that the bonding of R6G and ZnONPs was stable (Additional file 1: Fig. S2 B–D).

### ZnONPs exacerbate skin damage in mice after UVB irradiation

**Fig. 2:**
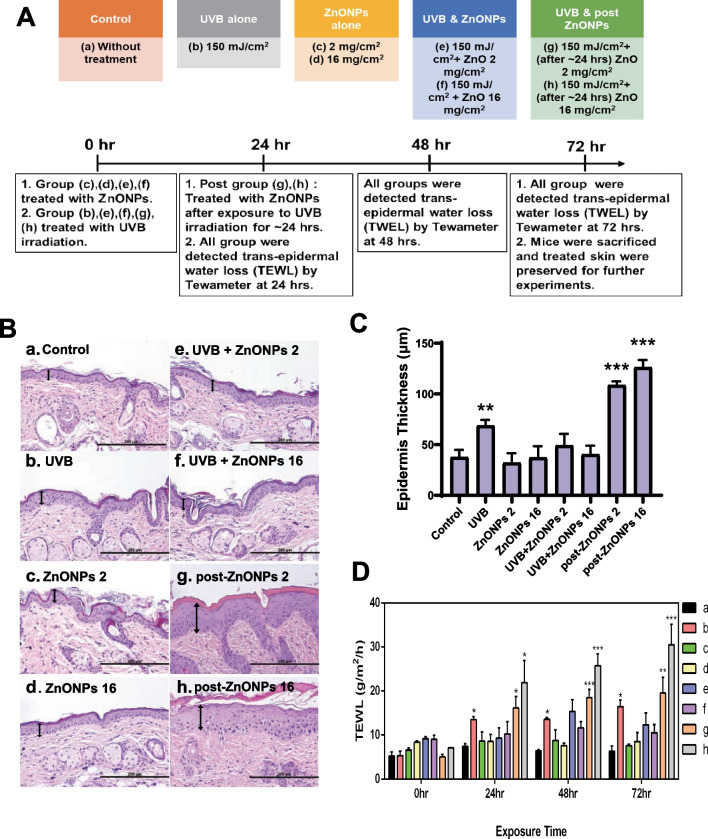

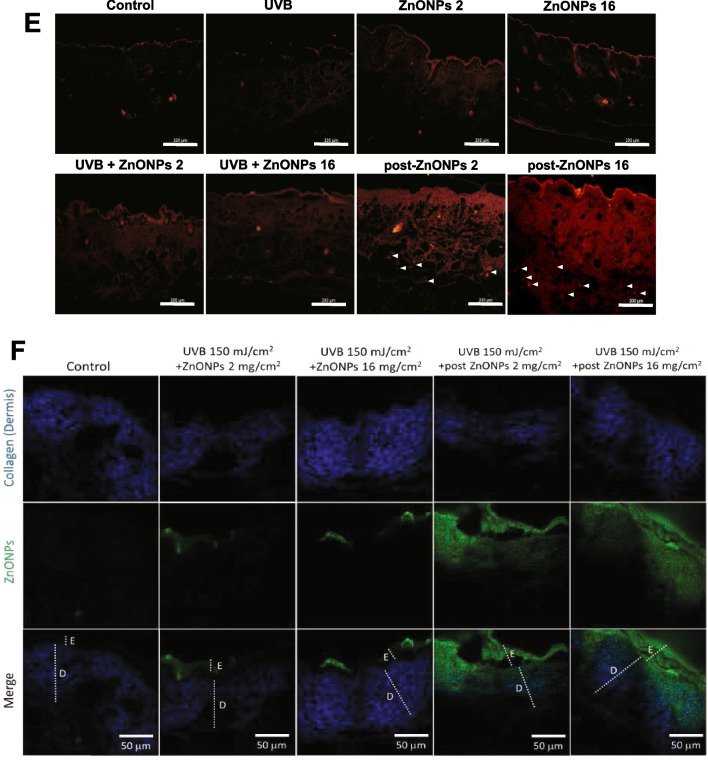
Skin lesions of UVB and ZnONPs exposure. **A** Description of the SKH:HR-1 mouse experimental groups and exposure methods. **B** Hematoxylin and eosin stains of skin on SKH:HR-1 mice. Skin thickness after treatment for 72 h. The double-headed arrow indicates the thickness of the mouse epidermis. Scale bars: 200 μm. **C** Histograms depict the measurement of epidermal thickness following various treatments. The data is presented as the mean ± SD from three independent experiments. (**p < 0.01, ***p < 0.001 compared with control). **D** TEWL of the skin of SKH:HR-1 mice were detected by Tewameter after exposure for 72 h. The values are presented as percentages relative to the TEWL without treatment. a: Normal skin (control), b: 150 mJ/cm^2^ UVB, c: 2 mg/cm^2^ ZnONPs, d: 16 mg/cm^2^ ZnONPs, e: 150 mJ/cm^2^ UVB + 2 mg/cm^2^ ZnONPs, f: 150 mJ/cm^2^ UVB + 16 mg/cm^2^ ZnONPs, g: 150 mJ UVB /cm^2^ + 2 mg/cm^2^ post ZnONPs, and h: 150 mJ/cm^2^ UVB + 16 mg/cm^2^ post ZnONPs. **E** Fluorescence photomicrograph of vertical slices of OCT compound of SKH:HR-1 mouse skin after the application of R6G-labeled ZnONPs for 72 h. UVB caused skin damage, enhancing the penetration of ZnONPs into the deeper skin layer. Scale bars: 200 μm. **F** Multiphoton fluorescence image showing ZnONPs (green fluorescence) and collagen (blue fluorescence) in untreated skin (control), skin treated with 2 or 16 mg/cm^2^ ZnONPs plus 150 mJ/cm^2^ UVB, and skin treated with 150 mJ/cm^2^ UVB combined with 2 or 16 mg/cm^2^ post ZnONPs for 72 h. E: epidermis, D: dermis, Scale bar: 50 μm

The animals were divided into 8 groups that were exposed to different doses (Fig. 2Aa–h). Except for the individual exposure groups, the combined exposure groups were simultaneously exposed or pre-exposed to UVB (150 mJ/cm^2^), and ZnONPs were applied to the dorsal skin after 24 h. The experimental involved observing whether the reaction of healthy skin or sunburned skin to ZnONPs was different. Two doses of ZnONPs (2 mg/cm^2^ and 16 mg/cm^2^) were applied to the mouse dorsal skin in the present study. A detailed description of the methods is shown in the supplementary figures (Additional file 1: Fig. S3). To test the skin damage and inflammation caused by ZnONP, UVB and combined exposure, we used SKH:HR-1 hairless mice as an animal model and analyzed the skin reactions of the mice at specific time points (24, 48 or 72 h after UVB exposure). We sought to evaluate the thickening of the epidermis by H&E staining. In Fig. 2B, the epidermal layer was pointed by double headed arrow. Results showed there was a slightly epidermal layer thickness in the UVB alone group (Fig. 2Bb). As expected, ZnONPs alone (Fig. 2Bc, d) did not directly affect the intact skin; therefore, the thickness of the epidermal layer was similar to that of the control group. The simultaneous exposure groups (Fig. 2Be, f) showed the same epidermal thickness results as the control. However, if mice were exposed to UVB in advance and then treated with ZnONPs (post-ZnONPs), significant thickening of the epidermis was observed (Fig. 2Bg, h). It could be clearly observed from the quantitative graph that the epidermal layer was increased 3 to 5 times compared with the control group and even more thickened than the UVB alone group (Fig. 2C). As the ZnONP dose increased, the thickness of the epidermal layer became more obvious. In general, healthy skin has normal skin functions due to its abundant moisture. Many dermatology or cosmetic studies are used to examine TEWL as an indicator of skin function. When TEWL becomes higher, the skin tissue loses more water and is more susceptible to slight external irritation. To evaluate the skin damage of different exposure groups, the TEWL of mouse skin was measured at 24, 48 and 72 h after UVB exposure (Fig. 2D). In normal skin conditions, the TEWL is approximately 5 g/m^2^/h, and the value remains stable under the conditions of constant temperature and humidity in the animal center. After UVB exposure (Fig. 2Db), an increase in TEWL was observed, indicative of UVB-induced sunburn. TEWL values exhibited a significant increase at 24 h, which was sustained at both 48 and 72 h. The highest value was approximately 15 g/m^2^/h. The TEWL values of the ZnONP alone groups were consistent with the H&E staining results, and there was no obvious TEWL effect on the skin (Fig. 2Dc, d). Simultaneous exposure groups showed that TEWL values slightly increased after 48 h of exposure, but the increase slowed down and returned to normal within 72 h (Fig. 2De, f). The post-ZnONP groups exhibited severe TEWL, which was 2 to 3 times higher than that of the control group. When exposed to a higher dose of ZnONPs (16 mg/cm^2^) after UVB exposure, there was a significantly increasing TEWL value compared with other exposure groups within 24 h, and its TEWL values may still increase even after 72 h (Fig. 2Dh). To confirm the penetration ability of ZnONPs into the skin, we observed the distribution of particles by fluorescently labeled ZnONPs in the skin (Fig. 2E). There were few fluorescent background values in the stratum corneum and hair follicles in the control group. In the case of single exposure to ZnONPs, it was obvious that many ZnONPs accumulated in hair follicles, causing red fluorescence on the skin surface and hair follicles. When the skin was damaged, ZnONPs could penetrate into the deeper skin layer, and we found that brighter red dots were distributed in the skin tissue (especially in the dermis). In addition, multiphoton microscopy was used to directly detect ZnONPs in skin sections. According to the fluorescence emission spectrum results (Additional file 1: Figure S1), ZnONPs could be identified by green fluorescence emissions. The collagen fibers in the dermis layer emitted a second harmonic generation (SHG) blue signal in the multiphoton microscope system. As shown in Fig. 2F, there was no green ZnONPs signal in the control group. When ZnONPs were applied to intact skin, green ZnONPs fluorescence was detected only in the epidermal layer. However, in UVB-damaged skin, green ZnONPs fluorescence could be detected in both the epidermis and dermis layers. The multiphoton detection and R6G-ZnONP detection results provide evidence that ZnONPs could penetrate deeper layers in damaged skin. These results indicated that UVB could destroy the skin barrier and make ZnONPs more easily reach deeper skin tissue and affect the system of skin repair.

### ZnONPs combined with UVB exposure causes more severe toxicity in HaCaT cells by inducing autophagy blockage and lysosome dysfunction

**Fig. 3:**
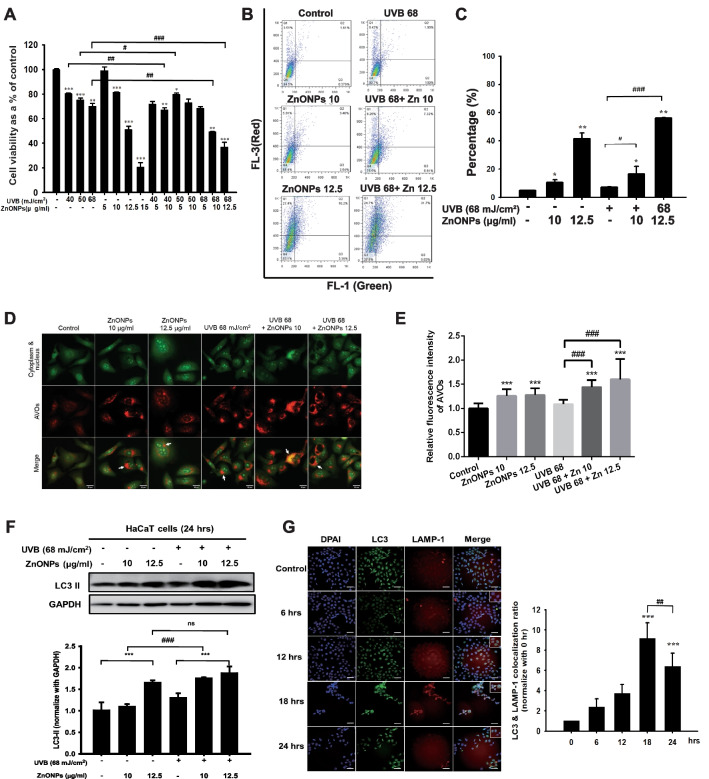

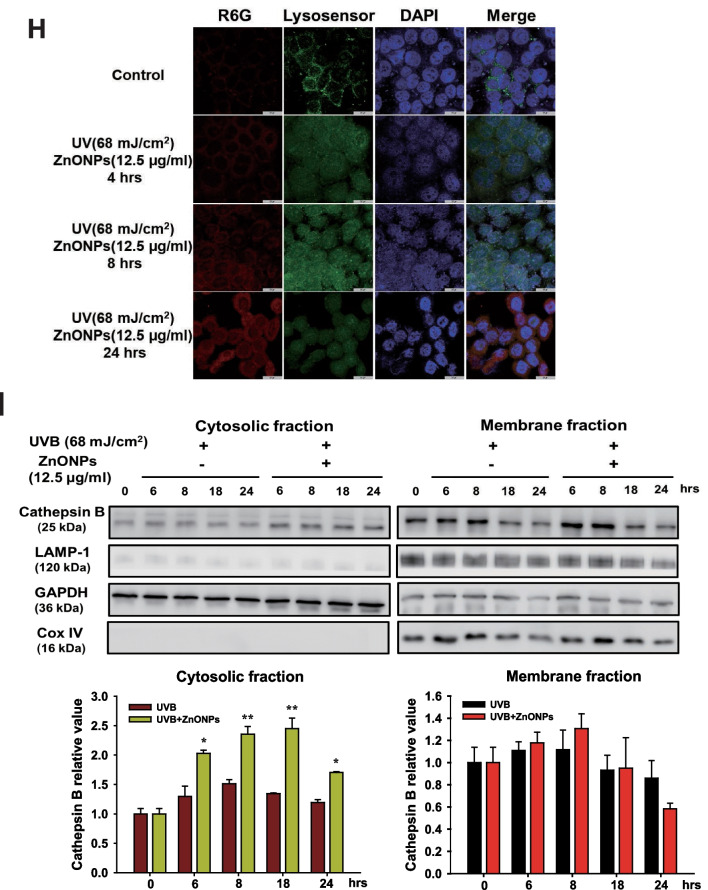
UVB and ZnONPs caused severe cell damage and subsequent lysosome dysfunction to induce autophagic flux blockage. **A** HaCaT cells were examined after 24 h using a trypan blue dye exclusion assay. Histograms show the percentages of in the control, UVB irradiation alone (40, 50, 68 mJ/cm^2^), ZnONPs alone (5, 10, 12.5, 15 μg/ml) and combined treatment groups. (*p < 0.05, **p < 0.01, ***p < 0.001 compared with control, #*p* < 0.05, ##*p* < 0.01, ###*p* < 0.001 compared with UVB alone) **B** ZnONPs, UVB and combination treatment induced autophagy in HaCaT cells at 24 h. Flow cytometry was used to detect green (FL-1) and red (FL-3) fluorescence in AO-stained cells. **C** Histograms show the percentages of autophagic cells after the different treatments. The data are presented as the mean ± SD from three independent experiments (*p < 0.05, **p < 0.01, compared with control, #*p* < 0.05, ###*p* < 0.001 compared with UVB alone). **D** Fluorescence microscope was used to visualize the acidic vesicular organelles (AVOs; red fluorescence) as well as the cytoplasm and nucleus (green fluorescence) after the AO staining. **E** Histogram represents the quantification of the AVOs red fluorescence (***p < 0.001, compared with control, ###*p* < 0.001 compared with UVB alone). **F** Measurement of autophagy-related proteins in HaCaT cells after UVB and ZnONP treatment for 24 h. **G** Immunofluorescence assays with LC3 (green) and LAMP-1 (red) antibodies were performed to identify the sites of autophagosomes and lysosomes. Scale bars: 50 μm. The fusion of autophagosomes with lysosomes was blocked when cells were exposed to ZnONPs for 24 h. **H** Fluorescence assays of R6G-labeled ZnONPs and lysosomal activity in the cells under a time course of combined treatment. Lysosomal activity was detected by LysoSensor DND-189 staining. Scale bars: 25 μm. DAPI staining showed the nuclear DNA of cells. **I** Cytosolic release of lysosomal proteases was detected by western blotting. HaCaT cells were treated with UVB alone and UVB + ZnONPs for the indicated times, followed by cell fractionation to obtain cytosolic and membrane fractions. LAMP-1 is a lysosomal membrane marker, and GAPDH is a cytosolic marker. There was no cross-contamination of cytosolic and lysosomal fractions when we separated the cell fractions. Cathepsin B was detected in the cytosolic fraction after 6 h of exposure to UVB + ZnONPs and was consistently expressed after 24 h of exposure

As the results of animal experiments indicated that combined exposure to UVB and ZnONPs caused more severe damage to the epidermal layer of the skin, we conducted an in vitro study to explore the related mechanisms. Keratinocytes are the most abundant cell types in the epidermis of the skin. In our study, we utilized HaCaT cells, human keratinocytes, to investigate the cytotoxicity of ZnONP, UVB, and combined exposure. As shown in Fig. 3A, UVB treatment alone (40, 50, and 68 mJ/cm^2^) resulted in a 75–80% cell survival rate, and ZnONP treatment alone had a concentration-dependent toxic effect on HaCaT cells, with a half-lethal concentration of ~ 12.5 μg/ml. In the combined treatment, we found that there was a significant concentration-dependent toxic effect when ZnONPs were combined with 68 mJ/cm^2^ UVB exposure. ZnONPs (10 μg/ml) combined with 68 mJ/cm^2^ UVB caused ~ 50% cell death; thus, we chose 10 and 12.5 μg/ml ZnONPs combined with 68 mJ/cm^2^ UVB for further investigation. Previous studies have reported that ZnONPs can induce autophagy via oxidative stress with the accumulation of autophagosomes and impairment of autophagic flux [34, 35]. We sought to evaluate the autophagy levels in the ZnONP alone, UVB alone, and combined exposure groups. Flow cytometry revealed that ZnONPs significantly increased AO-positive cells, and 12.5 μg/ml ZnONPs combined with 68 mJ/cm^2^ UVB induced more autophagy than UVB and ZnONPs exposure alone (Fig. 3B & C). Fluorescence microscope was also used to detect the acidic vesicular organelles (AVOs) in the cytoplasm. The measurement of red fluorescence of AO showed the similar pattern with flow cytometry results (Fig. 3D & E). This alignment was further substantiated by the detection of the autophagy-specific protein LC3 II (Fig. 3F). LC3 II protein accumulated when HaCaT cells were exposed to ZnONPs (12.5 μg/ml) or combined UVB exposure. Evidence suggests that nanomaterials induce autophagy flux impairment. The disturbance of autophagy flux via interference with lysosomal stability is one of the major reasons for autophagy blockage, which changes the fusion of autophagosomes with lysosomes and results in the accumulation of LC3 II protein [36]. Therefore, we observed the time course of LC3 and LAMP-1 (which is lysosome-associated membrane glycoprotein 1) protein expression and quantified their colocalization ratio from combined exposure cells. Interestingly, combined exposure caused the LC3 and LAMP-1 colocalization ratio to increase at 18 h and was significantly reduced at 24 h (Fig. 3G). These results indicated that combined exposure might induce autophagic flux defects in HaCaT cells. To further clarify whether the autophagy blockage was due to cellular uptake of ZnONPs and led to lysosome dysfunction, fluorescently labeled ZnONPs were used to observe the particles located in the cell, and we utilized LysoSensor staining to confirm lysosomal stability and activity (Fig. 3H). The fluorescence of the Lysosensor was increased in a time-dependent manner; however, the green positive fluorescent puncta became vague in the cytosol. The red fluorescence-labeled ZnONPs increased in cells, and the green fluorescence was degraded after 24 h of exposure, indicating that ZnONPs were taken up by cells, resulting in dysfunctional lysosomal storage, which may be caused by the loss of acidic conditions in the lysosome. In general, damage to lysosomes will increase lysosomal membrane permeabilization (LMP), resulting in the release of lysosomal contents, including proteolytic enzymes of the cathepsin family, into the cytoplasm [37]. The cathepsin family, such as cathepsin B, normally resides within the lysosomal lumen, and upon LMP, cathepsin B can be released into the cytosol, triggering cell death [37, 38]. To verify the level of LMP after exposure to UVB or combined exposure, we performed cell fractionation, followed by western blotting, to detect cathepsin B in cytosolic fractions of cells at different time points (Fig. 3I). As shown in the cytosolic fractions, the level of cathepsin B increased with exposure time to the combined treatment, consistent with the progressive lysosomal damage observed in the fluorescent image in Fig. 3H. In contrast, UVB alone did not show an increasing level of cathepsin B in the cytosolic fractions, indicating that cells may trigger cellular protective mechanisms at this UVB dosage; however, when cells were exposed to UVB and ZnONPs, it caused irreversible cell damage by disrupting autophagic flux and lysosome stability.

### HaCaT cells secrete exosomes to recruit THP-1-derived macrophages (MØs)

**Fig. 4:**
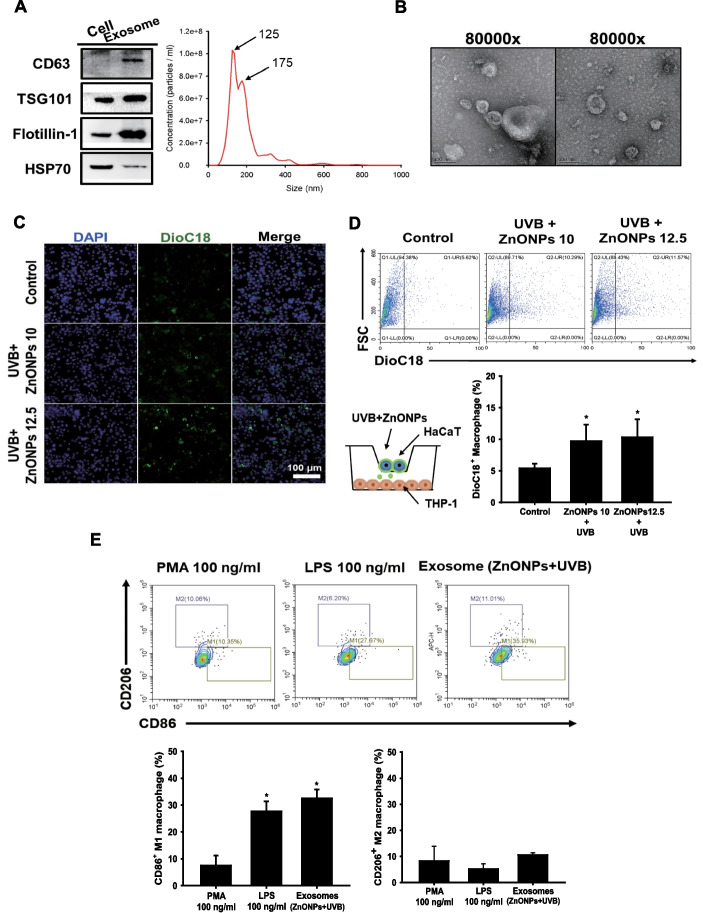

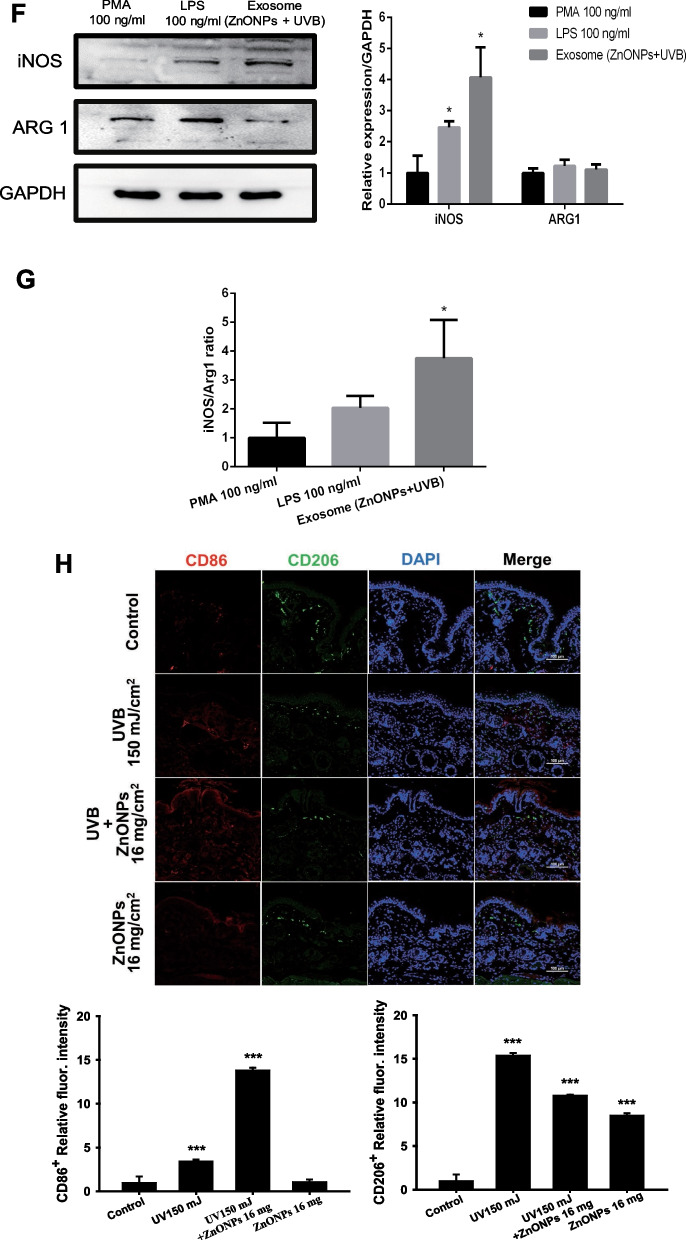
M1/M2 macrophage polarization was changed by treatment with exosomes (UVB + ZnONPs) from HaCaT cells. **A** Characterization of HaCaT cell-secreted exosomes. Exosomes were isolated from HaCaT cell culture medium, and exosome markers, including CD63, TSG101, and Flotillin-1, were identified by western blotting. The number of particles isolated from HaCaT cells was determined by nanoparticle tracking analysis (NTA). **B** Representative TEM images of exosomes obtained from untreated HaCaT cells (scale bar represents 100 nm). **C** DioC18 fluorescence-labeled HaCaT cells and THP-1-derived macrophages were cocultured in a transwell manner, as depicted schematically. Representative data showing the presence of DioC18-labeled exosomes that were internalized by macrophages after HaCaT cells were exposed to UVB + ZnONPs for 24 h (scale bar represents 100 μm). **D** Flow cytometry showed an increase in the amount of fluorescence-labeled exosomes released and taken up by macrophages in the UVB + ZnONP-treated group (*p < 0.05 compared with control). **E** Exosomes purified from HaCaT cells exposed to UVB + ZnONPs were added to THP-1-derived macrophages for 24 h. An increase in the M1 macrophage population was observed by flow cytometry when THP-1-derived macrophages were exposed to LPS (100 ng/ml) and exosomes (UVB + ZnONPs). PMA stimulates THP-1-derived macrophages. To mitigate the potential impact of PMA on macrophage polarization, we employed PMA as an internal control. (*p < 0.05 compared with control). **F** Western blot analysis was conducted to assess the protein expression of inducible nitric oxide synthase (iNOS, M1) and arginase-1 (ARG1, M2) in PMA-derived macrophages, LPS (100 ng/ml), and exosomes (UVB + ZnONPs). The histograms illustrate the relative expression levels of iNOS and ARG1 (*p < 0.05 compared with control). **G** The iNOS/ ARG1 ratio in the LPS and exosome exposure groups was normalized to that of the PMA group. (*p < 0.05 compared with control). **H** Mouse skin biopsies were collected from the different experimental groups after treatment for 72 h. Immunofluorescence analysis of CD86 (M1 macrophage marker) and CD206 (M2 macrophage marker) was performed to distinguish the M1/M2 macrophage populations (scale bar represents 100 μm). The quantification of CD86^+^ and CD206^+^ cells was evaluated by ImageJ software (***p < 0.001 compared with control). DAPI staining labeled the nuclear DNA of cells

The roles of exosomes in physiology and disease have drawn researchers’ attention during the past decade. Through regulating cellular exosome biogenesis, it has been proven to influence cell proliferation, apoptosis, cytokine production, and the immune response [27]. In the present study, we determined that combined exposure caused the disturbance of autophagy flux and lysosome rupture; thus, exosome biogenesis may be affected and interfere with cell‒cell interactions [39]. To elucidate the effect of exosomes released from keratinocytes on macrophages under combined exposure, we first collected exosomes secreted by HaCaT cells and identified their characteristics. In Fig. 4A, CD63, TSG101, Flotillin-1, and heat-shock 70-kDa proteins were used as exosome markers. The results showed that we successfully separated exosomes from the culture medium, and the major sizes of the exosomes were 125 nm and 175 nm. TEM images presented the morphology of exosomes, and there were clear cup-shaped exosomes in the collected samples (Fig. 4B). To track whether the exosomes released by HaCaT cells were internalized by macrophages, we used DioC18 dye to label HaCaT cells and cocultured HaCaT cells and THP-1-MØs in a transwell plate. As shown in Fig. 4C, there were significant increases in DioC18-labeled exosomes internalized by macrophages when treated with UVB + ZnONPs. The increased internalization of HaCaT cell-secreted exosomes was also detected and quantified by flow cytometry (Fig. 4D). These results showed that injured HaCaT cells may transfer cellular messages by exosomes to regulate macrophage activity; therefore, we further explored what kinds of effects occur when these exosomes enter macrophages. When THP-1-MØ were treated with exosomes, which were derived from HaCaT cells exposed to UVB + ZnONPs, the expression of the M1 surface marker CD86 increased compared to the PMA (phorbol-12-myristate-13-acetate) control, and the M2 surface marker CD206 had no significant changes (Fig. 4E). Western blot analysis was also used to examine the relative expression of iNOS (M1 marker) and ARG1 (M2 marker), THP-1-MØ treated with UVB + ZnONPs exosome significantly increased the expression of iNOS while ARG1 remained unchanged (Fig. 4F). The ratio of iNOS and ARG1 was significantly increased (Fig. 4G). These data suggest that THP-1-MØs exposed to UVB + ZnONPs exosome tended to be polarized to an M1-like phenotype and promoted inflammatory responses. Normally, signal transducers and transcriptional activators affect macrophage polarization; for example, lipopolysaccharide (LPS) initiates the NFκB transcription factor to increase M1 macrophage polarization [13, 40]. In skin biopsies, we also examined the populations of M1 and M2 macrophages by surface marker staining. The results showed a significantly increased M1 macrophage population in the combined exposure group (150 mJ/cm^2^ UVB + post-16 mg/cm^2^ ZnONPs) compared to the UVB alone group. Therefore, ZnONPs may play a similar role to LPS in sunburn skin conditions. In contrast, UVB alone tended to induce more M2 macrophages to limit excessive inflammatory responses and promote tissue repair (Fig. 4H) (Additional file 1: Fig. S4). These results might explain why ZnONPs caused more severe skin inflammation and extended skin water loss after UVB exposure in mouse skin (as shown in Fig. 2). In order to clarify whether ZnONPs is the active ingredient inside the exosomes. We used EDS-TEM mapping to identify the element composition inside the exosome. The EDS result indicated that the ratio of zinc within exosome is very low, therefore the probability of ZnONPs as an active ingredient seems unlikely (Additional file 1: Fig. S5).

### ZnONP penetration caused cellular stresses, inflammatory responses, and toxicity in THP-1-MØ

**Fig. 5:**
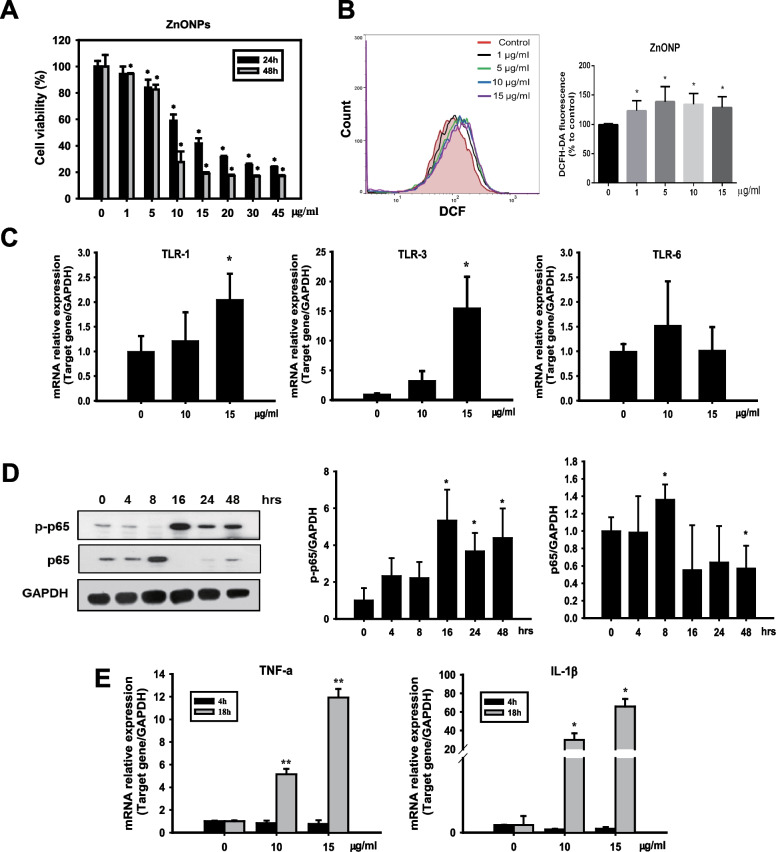

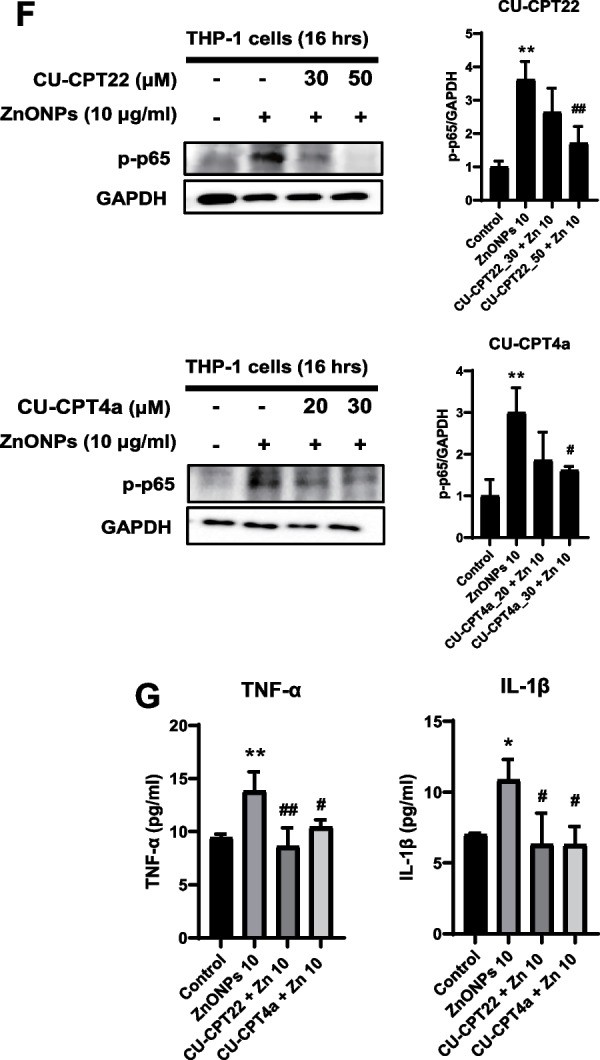
ZnONPs induced cytotoxicity and innate immune responses through Toll-like receptor (TLR) signaling in THP-1-derived macrophages. THP-1 cells were incubated for 24 h in the presence of 10 ng/ml PMA and then exposed to various concentrations of ZnONPs for different times. **A** Effects of ZnONPs on THP-1-derived macrophage viability after 24 or 48 h. Cell viability was measured by MTT assays. **B** ZnONP-induced ROS generation in THP-1-derived macrophages. After being exposed to ZnONPs, the cells were incubated with 10 μM DCFH-DA for 30 min to stain H_2_O_2_ and analyzed by flow cytometry. Histograms show the percentages of DCFH-DA fluorescence compared with the control. The data are presented as the mean ± SD from three independent experiments. (*p < 0.05, compared with control) **C** THP-1-derived macrophages were treated with ZnONPs (10 and 15 μg/ml) for 18 h, and the mRNA expression of TLR1, TLR3 and TLR6 was examined by RT‒qPCR and normalized to GAPDH expression. The data were presented as the mean ± SD from three independent experiments. The results showed that ZnONPs significantly triggered TLR1 and TLR3 activation. **D** THP-1-derived macrophages were treated with ZnONPs (10 μg/ml) for various times, and NFκB (p-p65) protein expression was analyzed by western blotting. **E** The mRNA levels of the proinflammatory cytokines TNF-α and IL-1β were determined by RT‒qPCR and normalized to GAPDH expression. The data are presented as the mean ± SD from three independent experiments. ZnONPs increased TNF-α and IL-1β mRNA expression at 18 h. (*p < 0.05, **p < 0.01 compared with control). **F** THP-1-derived macrophages were treated with ZnONPs (10 μg/ml), and CU-CPT22 or CU-CPT 4a for 16 h, then NFκB (p-p65) protein expression was analyzed by western blotting. CU-CPT22 and CU-CPT 4a are inhibitors specific to TLR1/2 and TLR3, respectively. **G** Cells were treated with ZnONPs (10 μg/ml), and CU-CPT22 (50 μM) or CU-CPT 4a (30 μM) for 24 h. TNF-α and IL-1β protein levels in the culture medium were measured by ELISA. The data were presented as the mean ± SD from three independent experiments. (*p < 0.05, **p < 0.01 compared with control, #*p* < 0.05, ##*p* < 0.01 compared with ZnONPs alone)

The abovementioned data showed penetration of ZnONPs through UVB-damaged skin into the dermis (Fig. 2E and F). Therefore, we further investigated the effects of pervading ZnONPs by taking advantage of THP-1-MØ cells to observe ZnONP-induced cell toxicity and the inflammatory response. In the cytotoxicity study, we treated THP-1-MØ cells with ZnONPs and found a significant decrease in cell viability in a time- and concentration-dependent manner (Fig. 5A). Additionally, ROS (Reactive oxygen species) accumulation was detected in the cells exposed to ZnONPs (Fig. 5B). Since ZnONPs were evident to cause the cell death of macrophages and modulate the innate immune responses by activating Toll-like receptors (TLRs) [41, 42], we examined the expression of TLRs in macrophages with ZnONP induction. The results demonstrated that ZnONPs induced the mRNA expression of TLR-1 and TLR-3 but not TLR-6 in macrophages. This finding suggested that ZnONP-induced stress was sufficient to activate the proinflammatory pathway via TLRs, which are expressed in cell membranes and endosomes (Fig. 5C). The accumulated phosphorylated p65 (p-p65) signals also indicated an active immune response under ZnONP-induced cellular toxicity (Fig. 5D). Furthermore, we detected cytokine mRNA levels in stressed THP-1-MØs and found significant expression of TNF-α and IL-1β mRNA after 18 h of exposure to ZnONPs (Fig. 5E). Additionally, we employed TLR1/2 inhibitor (CU-CPT 22) and TLR3 inhibitor (CU-CPT 4a) to substantiate the involvement of TLR signaling in the inflammatory activation of macrophages. The findings revealed that CU-CPT22 and CU-CPT 4a effectively suppressed the ZnONPs-induced production of p-p65, TNF-α, and IL-1β (Fig. 5F and G). TLRs are a critical component of the innate immune system. TLRs are largely classified into two subfamilies based on their localization: cell surface TLRs and cytoplasmic TLRs [43]. Triggering TLRs is one of the canonical pathways to activate the transcription factors nuclear factor-κB (NF-κB) and interferon regulatory factors (IRFs), which dictate the outcome of innate immune responses and promote the induction of inflammatory cytokines [21, 44]. In the present study, we showed that both cell surface and cytoplasmic TLRs were elevated by ZnONPs, especially TLR1 and TLR3, which initiated the NF-κB signaling pathway to induce the synthesis of TNF-α and IL-1β, causing further inflammation.

### ZnONPs disturbed autophagic flux, causing NLRP3 inflammasome activation in THP-1-MØs

**Fig. 6:**
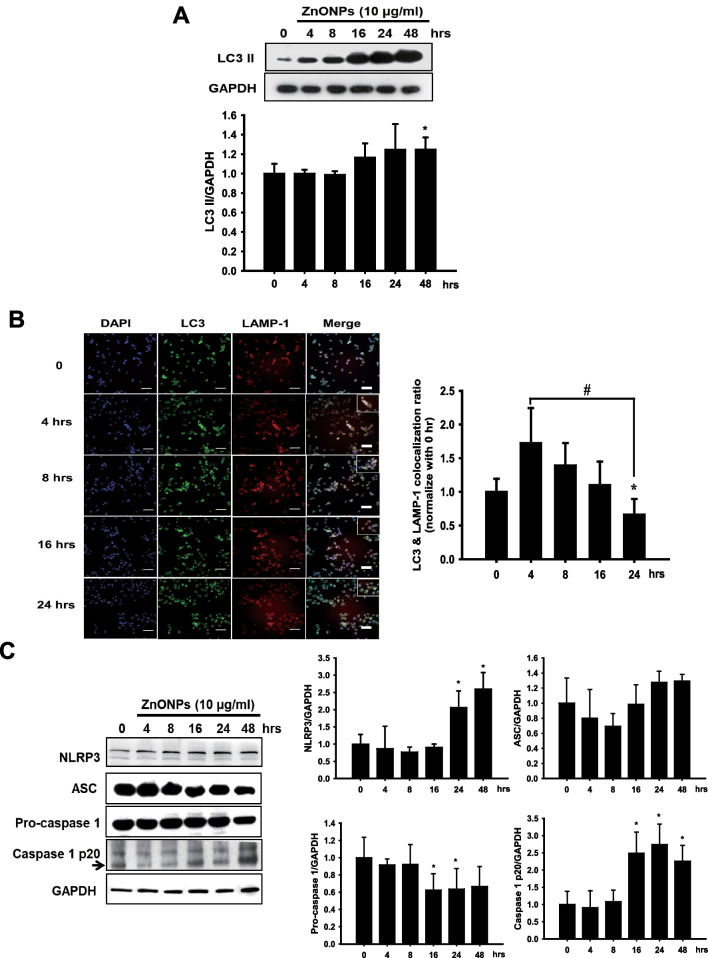

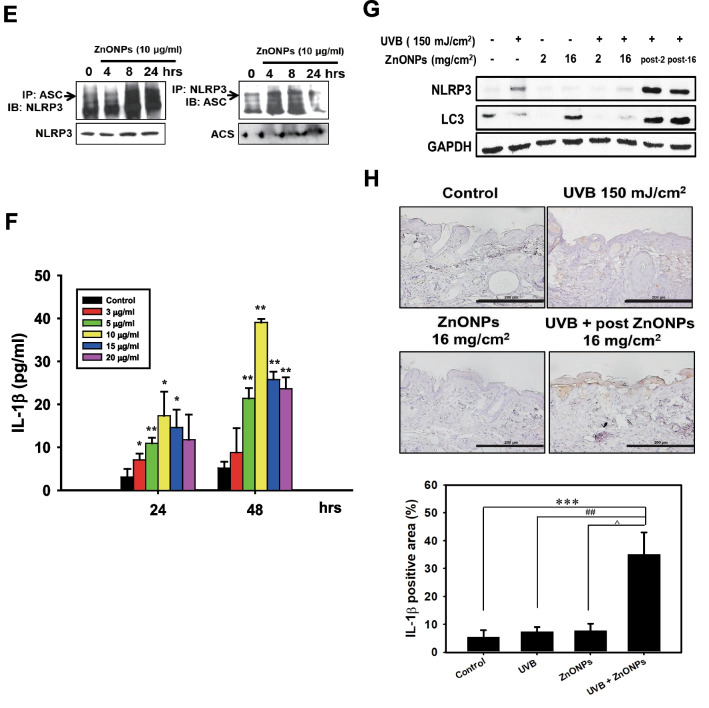
ZnONPs obstructed the fusion of autophagosome with lysosome and caused NLRP-3 inflammasome activation leading to IL-1β expression and prolonged skin inflammation. THP-1 cells were differentiated with PMA for 24 h and then exposed to 10 μg/ml ZnONPs for various times. **A** After being exposed to ZnONPs, THP-1- derived macrophage proteins were extracted, and LC3 II protein expression was assessed by western blotting. GAPDH was used as a loading control. Histograms show the quantification of LC3 II normalized to GAPDH in three independent experiments. The results were expressed as the mean ± SD. **B** Immunofluorescence assays with LC3 (green) and LAMP-1 (red) antibodies were performed to identify the location of autophagosomes and lysosomes. Scale bars: 50 μm. The fusion of autophagosomes with lysosomes was blocked when cells were exposed to ZnONPs for 24 h. **C** NLRP3, ASC and Caspase-1 protein expression was analyzed by western blotting, and GAPDH was used as a loading control. Histograms representing the quantification of NLRP3, ASC, caspase-1, and caspase-1 (p20) normalized to GAPDH in three independent experiments. The results were expressed as the mean ± SD. **D** Analysis of NLRP3 and ASC protein interactions by immunoprecipitation at various time points. **E** Cells were treated with various concentrations of ZnONPs for 24 h. IL-1β levels in the culture medium were measured by ELISA. The data were presented as the mean ± SD from three independent experiments. (*p < 0.05, **p < 0.01 compared with control). **F** Mouse skin proteins were collected from different experimental groups after treatment for 72 h. NLRP3 and LC3 protein expression was analyzed by western blotting, and GAPDH was used as a loading control. **G** Frozen skin sections were collected from the different experimental groups after treatment for 72 h and stained with an antibody against IL-1β. The expression of IL-1β was determined by immunohistochemistry. Scale bars: 200 μm

Autophagy has been proven to inhibit the activation of the NLRP3 inflammasome, which can be regarded as an anti-inflammatory defense mechanism [21, 44]. A previous study showed that rare-earth nanoparticles (REOs) can affect the formation of autolysosomes, which in turn leads to an increase in the NLRP3 inflammasome in macrophages and affects subsequent inflammation-related cytokine expression [45]. Therefore, the disruption of autophagy and triggering of the NLRP3 inflammasome is likely to induce proinflammatory cytokine mRNA expression when THP-1-MØs are exposed to ZnONPs. To confirm our point of view, we examined the expression of autophagy-related proteins and observed the fusion of autophagosomes with lysosomes. Our data indicated that ZnONP exposure resulted in LC3 II protein accumulation in a time-dependent manner (Fig. 6A). We next utilized lysosomal membrane markers, LAMP-1, and LC3 antibodies to identify the locations of autophagosomes and lysosomes in THP-1-MØs. After exposure to ZnONPs, cells presented more LC3-positive punctuate structures (green) than untreated cells. The LC3 and LAMP-1 (red) colocalization ratio increased at 4 h and was significantly reduced at 24 h (Fig. 6B). As shown in an enlarged view of the merged image, there were separate LAMP-1 (red) and LC3 (green) punctate structures at 24 h. We further investigated whether ZnONPs enter THP-1-MØs and cause dysfunctional lysosome activity. As shown in Additional file 1: Figure S7, R6G-ZnONPs were internalized by THP-1-MØ. However, over time, lysosomal activity (green) decreased, and R6G-ZnONPs (red) still accumulated in THP-1-MØ cells, which suggested that the ZnONPs indeed affected lysosomal activity to interfere with normal autophagic flux. Because ZnONPs induced autophagy dysfunction in THP-1-MØs, we examined NLRP3 inflammasome complex proteins and IL-1β cytokine expression in THP-1-MØs and skin biopsies. Activation of the NLRP3 inflammasome was performed in the cells under ZnONP exposure. The levels of NLRP3 and cleaved caspase-1 protein were increased. Furthermore, the interaction between NLRP3 and ASC was increased when THP-1-MØs were exposed to 10 μg/ml ZnONPs at various time points (Fig. 6C and D). The release of the proinflammatory cytokine IL-1β was also detected after ZnONP exposure (Fig. 6E), and the extraction of proteins from skin tissue showed greater increases in NLRP3 and LC3 protein levels in both post-ZnONP groups than in the UVB alone group (Fig. 6F). Interestingly, exposure to only a high dose of ZnONPs alone (16 mg/cm^2^) slightly increased LC3 protein levels but did not induce NLRP3 inflammasome activation, which suggested that it should be harmless when people apply the appropriate dose of ZnONPs in intact skin. In Fig. 6G, we examined the expression of IL-1β in the mouse skin epidermis. The results demonstrated that the post-ZnONP group had significant IL-1β protein expression even after 72 h of exposure but not in the UVB alone or ZnONP alone groups, which indicated that ZnONPs prolonged skin inflammation after UVB exposure.

### Exosomes derived from THP-1-MØ induced NLRP3 inflammasome activation in HaCaT cells

**Fig. 7:**
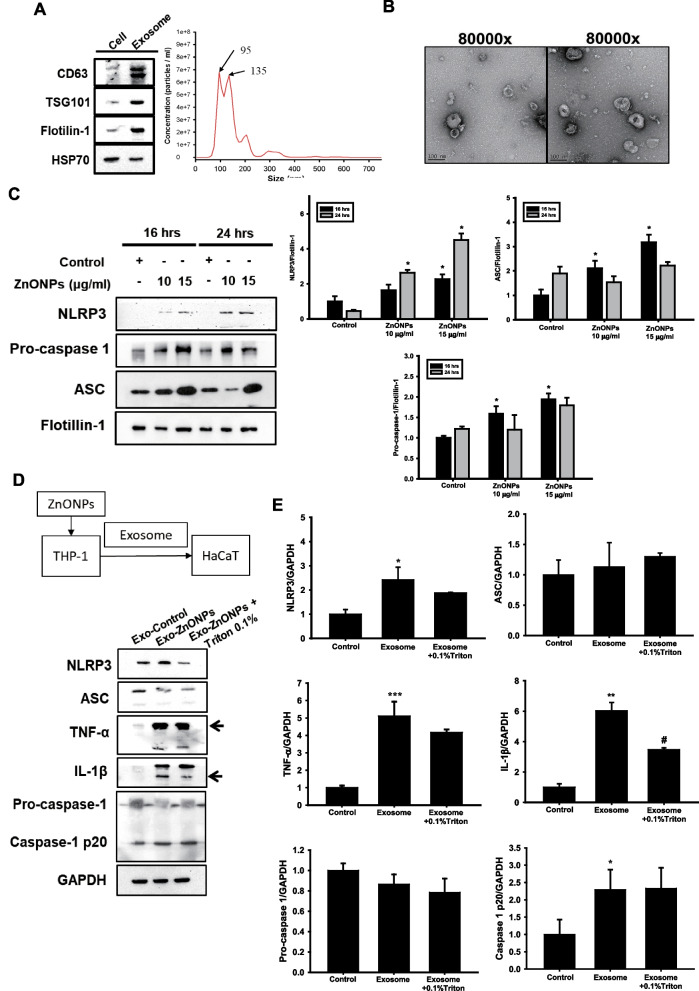
Exosomes containing NLRP3, ASC and Caspase-1 proteins were increased when THP-1-MØ cells were exposed to ZnONPs. Exo-ZnONPs from THP-1-MØ triggered NLRP3 inflammasome-related protein and proinflammatory cytokine expression in HaCaT cells. **A** Characterization of THP-1-derived macrophage-secreted exosomes. Exosomes were isolated from the macrophage culture medium, and exosome markers, including CD63, TSG101, and Flotillin-1, were identified by western blotting. The number of particles isolated from the culture medium was determined by NTA. **B** Representative TEM images of exosomes obtained from untreated macrophage culture medium. Scale bars: 100 nm. **C** THP-1-derived macrophages were exposed to ZnONPs (10 and 15 μg/ml) for 16 or 24 h. The exosomes in the different groups were extracted, and the exosome cargo proteins NLRP3, ASC, and Pro-caspase-1 were assessed by western blotting. Flotillin-1 was used as a loading control. Histograms representing the quantification of NLRP3, ASC, and pro-caspase-1 normalized to flotillin-1 in three independent experiments. The results were expressed as the mean ± SD. **D** HaCaT cells were incubated with macrophage-derived Exo-control, Exo-ZnONPs, and Exo-ZnONPs + Triton 0.1% for 24 h. HaCaT cells were extracted, and the expression of the NLRP3 inflammasome-related proteins TNF-α and IL-1β was assessed by western blotting. GAPDH was used as a loading control. **E** Histograms represent the quantification of NLRP3, ASC, caspase-1, caspase-1(p20), TNF-α, and IL-1β normalized to GAPDH in three independent experiments. The results are expressed as the mean ± SD. (*p < 0.05, **p < 0.01, ***p < 0.001 compared with control)

It has been suggested that lysosomes play a role in the regulation of exosome biogenesis and release [46]. Therefore, ZnONP-induced lysosomal impairment may change the secretion of exosomes derived from THP-1-MØs, which could initiate a variety of different inflammatory signals and influence neighboring cells. We collected exosomes derived from THP-1-MØs and examined their protein markers to confirm exosome purity. In Fig. 7A, CD63, TSG101, Flotillin-1, and heat-shock 70-kDa proteins were present in our separated exosomes, and the major sizes of the exosomes were 95 nm and 135 nm. TEM images showed the morphology of exosomes with a clear cup shape in the collected samples (Fig. 7B). In addition, we detected accumulation of NLRP3, pro-caspase 1, and ASC proteins in the exosomes derived from THP-1-MØ cells exposed to ZnONPs (10 and 15 μg/ml) after 16 or 24 h (Fig. 7C), which suggested that the NLRP3-inflammasome complex proteins were packaged by exosomes and then trafficked to neighboring cells or, alternatively, distal recipient cells for further biological responses. To examine whether ZnONP-induced exosomes from THP-1-MØ could mediate inflammation of HaCaT cells, we treated HaCaT cells with exosomes derived from THP-1-MØ with the control media (Exo-control), ZnONPs (Exo-ZnONPs), or Exo-ZnONPs plus 0.1% Triton X-100, which is normally used to breakdown membrane structure leading to exosome component release. After 24 h of stimulation, the Exo-ZnONPs increased NLRP3, cleaved caspase-1, TNF-α, and IL-1β protein levels compared to the Exo-control. When we broke down the Exo-ZnONPs membrane, the NLRP3 protein was inhibited, which alleviated IL-1β expression in HaCaT cells (Fig. 7D and E). Collectively, the findings showed that ZnONPs triggered THP-1-MØ release of exosomes and that Exo-ZnONPs containing NLRP3-inflammasome complex proteins were able to promote the inflammatory response of HaCaT cells to prolong skin epidermal injury.

## Discussion

The sun is essential for life on earth; however, excessive sunlight exposure has been indicated to cause sunburn, photoaging, melanoma, and nonmelanoma skin cancers [47]. A previous study indicated that indoor-working adults receive 10,000–50,000 mJ/cm^2^ ultraviolet radiation (UVR) per year (27.4–137 mJ/cm^2^ per day), and outdoor workers can receive three times higher UVR doses (82.2–411 mJ/cm^2^ per day) than indoor workers [48]. Acute and chronic exposure to UVR generally causes skin damage. Lavker et al. reported that daily exposure to 0.049 J/cm^2^ UVB and 8.053 J/cm^2^ UVA for 28 days increased the stratum corneum thickness and the depletion of Langerhans cells [49]. Human trials demonstrate that exposing the skin of seven healthy male volunteers (skin type II) to solar-simulated UV radiation at doses equivalent to 0, 3, and 6 standard erythemal doses (SED) led to changes in gene expression. This included genes associated with DNA repair and apoptosis, immunity and inflammation, pigmentation, and vitamin D synthesis [50].

To prevent harmful ultraviolet (UV) radiation, sunscreen has become a must-use cosmetic product in our daily life. The ingredients of sunscreens are quite diverse, and one of the main inorganic ingredients used to reflect UV radiation is zinc oxide (ZnO). ZnO is a multifunctional material possessing antibacterial and deodorizing properties, but it always gives a white appearance to the skin when used in sunscreen. Recently, new sunscreen formulations such as nanolized ingredients have emerged as suitable ways to solve these problems and improve sunscreen efficiency [51]. Indeed, regarding the safety issue of cosmetic ingredients, the Scientific Committee on Consumer Safety (SCCS) has published a number of scientific opinions in the past few years on the nanoforms of different materials. ZnONPs can be used in commercial skin products, such as sunscreens, based on the available evidence that ZnONPs at a concentration up to 25% as a UV filter in sunscreens is considered not to pose a risk of adverse effects in humans after dermal application (SCCS/1489/12 revision of 11 December 2012).

In the current study, we utilized SKH:HR-1 hairless mice for experimental purposes. Despite the heightened susceptibility of hairless mice to UVR-induced skin cancer and the potential immunologic and/or tissue homeostasis defects observed in certain strains, SKH:HR-1 mice remain a widely used animal strain in dermatological research. This includes investigations into wound healing, carcinogenesis, and toxicity [52]. Our in vivo experiment showed that ZnONPs alone did not affect the skin barrier or increase TEWL. However, when ZnONPs were applied to sunburned skin, they could not provide skin protection and even led to worse skin conditions (Fig. 2B–D). In order to prove our theory that ZnONPs could enter deeper layer of skin under sunburn condition. We used R6G-ZnONPs and multiphoton microscopy to detect R6G conjugate ZnONPs fluorescence and green fluorescence of ZnONPs which can excited by multiphoton laser without any fluorescence dye labeled. Although the R6G-labeled method showed several fluorescence background signals, which might be the autofluorescence by the skin section or the free R6G dye dissolved in the solution. The ZnONPs distribution of R6G-ZnONPs and ZnONPs showed same pattern in each group, and the XPS results indicated the R6G could form C-O bond on the surface of ZnONPs. Indicating the bonding of R6G and ZnONPs was stable and most R6G fluorescence represent the R6G-ZnONPs signal. These results indicated UVB could destroy the skin barrier, allowing ZnO NPs to more easily penetrate deeper skin tissues and potentially disrupt the system of skin repair. Therefore, the application of sunscreen containing nano-sized zinc oxide components on damaged or fragile skin may be inappropriate and should necessitate the provision of safety usage advisories.

Whether ZnONPs will penetrate into the deeper skin layer and cause harm has always been a topic of cosmetic safety when used in sunscreens. A recent study detected increasing zinc concentrations within the skin stratum in artificial human sweat after the application of ZnONPs to intact skin. However, when the skin barrier was impaired, there was a 60−65-fold increase in zinc concentrations within the epidermis compared to the intact skin control group [53]. Our current findings present evidence that, under UVB exposure conditions, ZnONPs exhibit enhanced penetration into the mouse skin, as illustrated in Fig. 2E and F. This determination was made utilizing indirect R6G-labeled ZnONPs and multiphoton microscopy. Additionally, existing research has indicated that TiO_2_ nanoparticles, also prevalent in sunscreens, possess the ability to permeate both intact and damaged skin. Furthermore, scientific evidence supports the entry of TiO_2_ nanoparticles and ZnONPs into the bloodstream, with detectable levels observed in human blood and urine. [54, 55]. While one study suggests that ZnO-NP does not induce NLRP3 inflammasome activation [56], the diverse physical–chemical properties of nanomaterials play a crucial role in determining distinct cellular toxicity. For example, surface modification of ZnONPs with SiO_2_ resulted in reduced enzyme leakage and lower oxidative stress, compared to bare ZnONPs [57]. Our previous study demonstrated that amino-modified (NH_2_-PS) polystyrene particles induce more pronounced toxicity than carboxylated (COOH-PS) modification [58].

In order to assess the applicability of our findings regarding ZnONPs toxicity, we employed another reference ZnONPs material (Sigma-Aldrich cat. 721,077) and examined various key events in this study, such as ROS generation, autophagy induction, and NLRP3 inflammasome activation. The results demonstrated that different types of ZnONPs can still elicit these crucial events at the cellular level (Additional file 1: Fig. S6), confirming the generality of our observation regarding ZnONPs-induced toxicity.

Our recent study indicated that ZnONPs can be taken up by HaCaT cells in the nano form and that coexposure to UVB and ZnONPs facilitates NLRP3 inflammasome activation and pyroptosis by disrupting cellular autophagy [33]. One of the related mechanisms to block autophagic flux is the fusion between autophagosomes and lysosomes. Several studies have shown that nanomaterials induce autophagy and cause autophagy dysfunction as their main biological effect [59]. Therefore, changes in lysosome activity or lysosomal integrity could regulate the clearance of autophagic cargo and decide the fate of cells. In the current work, our data demonstrated that ZnONPs were deposited within lysosomes and reduced lysosome activity by interfering with the pH of the lysosome internal environment in both HaCaT cells and THP-1-derived macrophages (Fig. 3H and Additional file 1: Fig. S7). The highly acidic environment maintains lysosome activity and the function of hydrolytic enzymes [60]. The internalization of ZnONPs into lysosomes may lead to the increased dissolution of zinc ions because of the acidic environment. Our data indicate that the lysosomal deposition of ZnONPs caused lysosomal membrane impairment, leading to the release of cathepsin B into the cytosol, the accumulation of undegraded autophagosomes, and subsequent cytotoxicity (Fig. 3G and I). A similar result has been observed in the context of neurotoxicity induced by ZnONPs. ZnONPs trigger cell death through the accumulation of autophagosomes, a process referred to as autophagic cell death [61].

Exosomes are nanosized extracellular vesicles originating from multivesicular endosomes (MVEs) and are released through fusion with the plasma membrane [62]. Interfering with endolysosomal trafficking has been proven to increase exosome production, which enhances the transport of intracellular bioactive molecules, including proteins, RNA, and lipids, to conduct cell‒cell communication [63]. A previous study suggested that exosomes play a pivotal role in nanoparticle-induced systemic immune activation. Evidence has shown that magnetic iron oxide nanoparticles induce exosome production in the alveolar region of mice, and these exosomes possess the ability to facilitate T-cell activation by promoting the differentiation of dendritic cells and macrophages [64]. Li et al. [65] found that exosomes from adipose-derived stem cells could regulate M1/M2 macrophage polarization to promote bone immune metabolism. In the present study, we found that HaCaT cell-secreted exosomes facilitate THP-1-MØ differentiation into an M1-like phenotype for a further inflammatory response (Fig. 4). In addition, THP-1-MØ treated with ZnONPs revealed the activation of the NLRP3 inflammasome complex in cells and stimulated exosome release from THP-1-MØ to influence the function of HaCaT cells involved in the prolonged inflammatory response (Fig. 7). However, our data suggest that ZnONPs is not the active ingredient inside the exosomes (Additional file 1: Fig S5). The secretory process of exosomes is complex. Accumulated evidence has revealed that autophagy-related proteins contribute to exosome biogenesis [66, 67]. For example, activating autophagy can accelerate the fusion of multiple vesicle bodies (MVBs) and autophagosomes, thereby inhibiting the release of exosomes from the erythroleukemia cell line [68]. In our current study, ZnONPs exacerbated UVB-induced autophagy dysfunction in HaCaT cells, and ZnONP exposure alone caused the same effect in THP-1-MØ cells. Therefore, the blockade of autophagic flux by ZnONP exposure could be the critical route for regulating exosome biogenesis.

## Conclusions

**Fig. 8:**
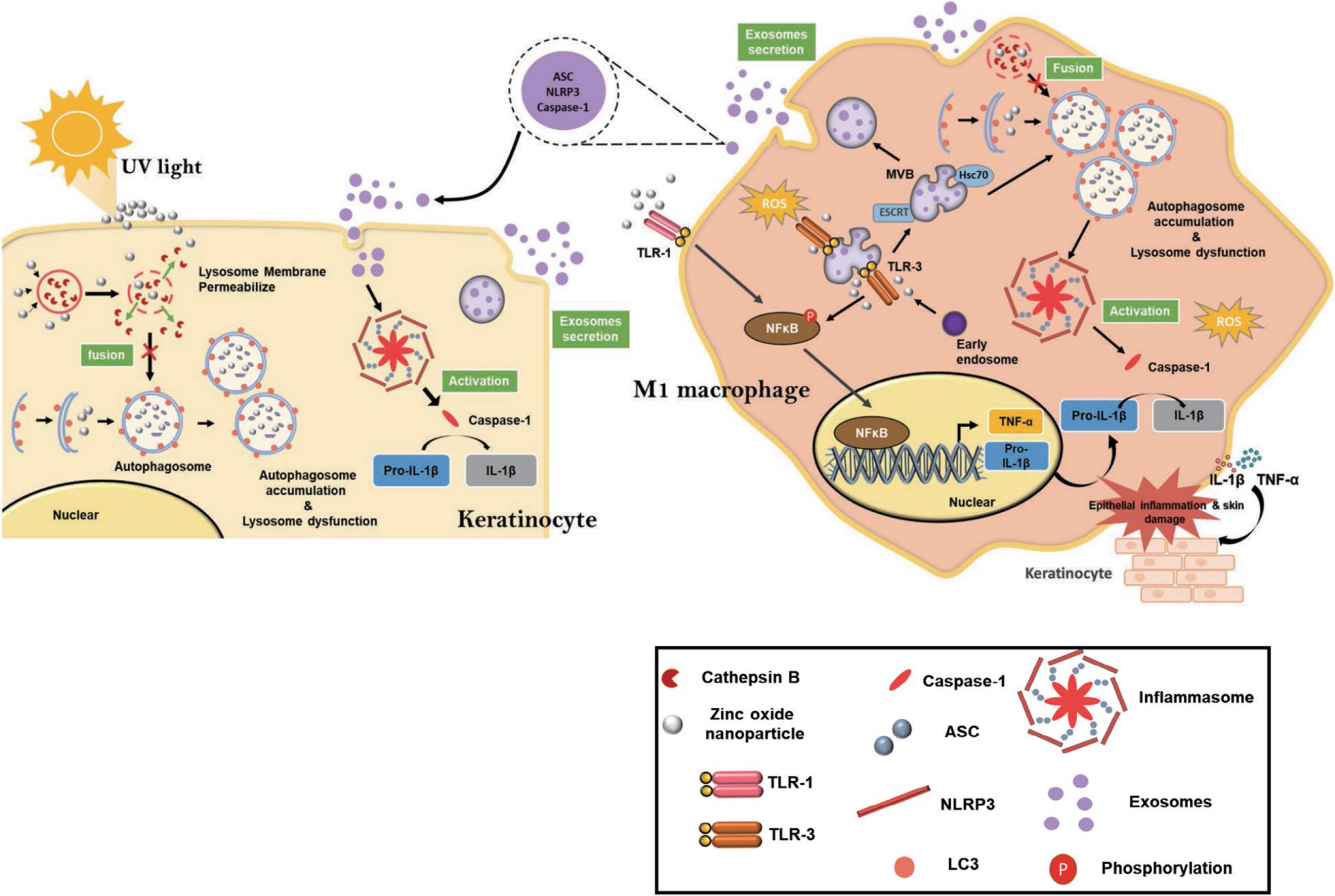
ZnONPs disrupt lysosome activity and impair autophagic flux to facilitate exosome secretion in keratinocytes and macrophages. Under UVB exposure, ZnONPs lead to autophagosome accumulation and lysosome dysfunction, which activate the NLRP3 inflammasome and enhance the release of exosomes by HaCaT cells. Moreover, exosomes secreted by HaCaT cells stimulated M1 macrophage activation, and further exposure to ZnONPs upregulated the NFκB signaling pathway and NLRP3 inflammasome-autophagy-exosomal pathway. Cytokines and exosomes released from macrophages further accelerated inflammatory responses in keratinocytes, which affected UVB-induced skin injury

In our present study, we identified potential deleterious effects of ZnONPs on compromised epithelium by penetrating into the dermis. ZnONPs exacerbated UVB-induced keratinocyte damage by disrupting autophagic flux, leading to increased exosome secretion in HaCaT cells. Internalized by THP-1-derived macrophages, ZnONPs activated the NFκB pathway, amplifying the inflammatory response. Additionally, ZnONPs interfered with lysosomal activity, inducing autophagy dysfunction, and upregulated the NLRP3 inflammasome as well as exosome secretion in macrophages (Fig. 8). We observed a significant increase in exosome secretion in HaCaT cells treated with ZnONPs following UVB exposure. These exosomes were found to be internalized by macrophages, inducing M1 macrophage polarization. Moreover, direct exposure of THP-1-derived macrophages to ZnONPs led to enhanced exosome secretion. Interestingly, the cargo within these exosomes stimulated the NLRP3 inflammasome and pro-inflammatory cytokines when exposed to HaCaT cells. Our findings highlight the crucial role of exosomes in mediating the toxicity induced by ZnONPs on both keratinocytes and macrophages, thereby influencing the repair of epithelial cells following UVB exposure. However, the limitation of the study is that we did not confirm the function of exosomes through in vivo experimentation. Furthermore, aside from macrophages, other immune cells, such as dendritic cells or neutrophils, may influence the inflammatory response in sunburned skin exposed to ZnONPs. These aspects will be studied in our future research.

Several studies have suggested that extracellular vesicles (EVs), including exosomes, can contribute to the progression of inflammatory diseases by inducing M1-like polarization of macrophages. Clinical interventions targeting EVs have become a prominent focus of current research. Therefore, further investigation into the cargos of exosomes derived from ZnONP-stimulated cells or targeting pathways regulating EVs secretion provides a promising direction for future research in the safety evaluation of ZnONPs or other nanomaterials used in skincare products. Collectively, the present study introduces a novel concept regarding the mechanisms of ZnONP-induced skin toxicity and the safety considerations associated with the application of ZnONPs, specifically on compromised skin rather than intact skin.

## Methods

### Materials

Dulbecco’s modified Eagle’s medium (DMEM), RPMI 1640 medium, penicillin, and streptomycin were purchased from Gibco BRL (Grand Island, NY, USA). Dimethyl sulfoxide (DMSO), PMA and LPS was purchased from Sigma Chemical Co. (Poole, Dorset, UK). CU-CPT22 and CU-CPT4a were purchased from MedChemExpress (NJ, USA).

Female hairless, nonpigmented mice (Crl:Skh1- Hr^hr^/Hr^hr^), referred herein as SKH:HR-1 mice, were a gift from Tzu-Kai Lin, M.D. (Department of Dermatology, College of Medicine, National Cheng Kung University, Taiwan), who obtained them from Charles River Laboratories (Wilmington, MA). SKH:HR-1 mice with the *hr* hypomorphic allele, are widely used as a substitute for human skin to measure percutaneous drug penetration, UVR irradiation damage, and wound healing.

Human keratinocyte HaCaT cells were a gift from Professor Hamm-Ming Sheu (Department of Dermatology, College of Medicine, National Cheng Kung University, Taiwan). The cells were cultured in DMEM (Gibco, NY, USA) supplemented with 10% fetal bovine serum (Gibco, NY, USA), 5% CO2 at 37 °C. Human monocyte THP-1 cells (BCRC #60,430) were obtain from the cell bank of Bioresource Collection and Research Center (BCRC).

### Preparation and Characterization of ZnONPs

The preparation of amine-modified zinc oxide nanoparticles (NH_2_-ZnONPs) was followed by previously published method [33]. Briefly, 3.35 mM zinc acetate dihydrate (Zn(CH_3_COO)_2_(H_2_O)) in 31.25 ml ethanol was mixed with potassium hydroxide (KOH) in 16.25 ml methanol and stirred at 60 °C. Then, KOH was added into the mixture and reacted for 1.5 h until the solution became turbid. The precipitate was collected by centrifugation at 10,000 rpm for 10 min, and then the pellet (ZnONPs) was washed twice with ethanol. Next, 0.25 ml APTES, 0.05 ml 25 wt% ammonia and 0.5 ml distilled water were added to the ZnONPs solution and stirred for 20 h at room temperature. The precipitate was collected by centrifugation at 10,000 rpm for 15 min. The resulting solution was the NH_2_-ZnONPs solution.

The size and morphology of ZnONPs was observed by using transmission electron microscope (TEM, JEOL JEM- 2100F transmission electron microscope). More than 100 particles were measured to determine the mean size. The nanoparticles’ element is analysis by using Energy Dispersive X-ray Spectrometer (EDS) function of TEM. The hydrodynamic diameters, polydispersity index and zeta potentials of ZnONPs were measured by DelsaNano C Particle Size and Zeta Potential Analyzer (Beckman Coulter, USA). The fluorescence spectrum of ZnONPs were measured by SpectraMax iD5 multi-mode microplate reader (Molecular devices, CA, USA).

### In vivo* experiment (Acute UVB Irradiation)*

The in vivo acute UVB irradiation model was established as described in a previous study [69]. Female SKH:HR-1 mice were used in the study. The minimal erythemal dose (MED) of hairless mice was approximately 60 mJ/cm^2^. Therefore, to induce acute damage from UVB irradiation, the irradiation intensity was set at 2.5 MED (150 mJ/cm^2^) [8, 33]. ZnONPs was administered at doses of 2 mg/cm^2^ (low dose) and 16 mg/cm^2^ (high dose) based on the standard protocol for determining the sun protection factor (SPF) of sunscreen products (ISO24444:2019) [7, 8]. First, Mice were exposed with a single dose of UVB (150 mJ/cm^2^) by using UV Crosslinker (UVP CL-1000). The animal experiment was divided into eight groups, including UVB, ZnONPs alone exposure, and combined exposure. One of the combined exposure groups was simultaneous exposure, in which mice skin were topically treated with ZnONPs (2 or 16 mg/cm^2^) then exposed to UVB and the other one was 24 h after UVB exposure, the mice were topically treated with ZnONPs (2 or 16 mg/cm^2^) in the same area. More detailed experimental processes were shown in Fig. 2A. The transepidermal water loss (TEWL) was measured at 24, 48 and 72 h by Tewameter® TM 300 probe (Courage & Khazaka GmbH, Cologne, Germany) after the UVB treatment. Mice were sacrificed at the time points described in the results, the skin was immediately harvested for further analysis.

For histopathological analysis, skin samples were fixed with 4% paraformaldehyde in PBS, then dehydrated and paraffin-embedded for further analysis. The other skin samples were stored at − 80 °C before protein for other analysis.

### Skin Thickness Measurement

The detail skin thickness measurement was described in previous study [33]. Briefly, the microscope image of Hematoxylin and eosin stained skin samples were used to determine the epidermis thickness then analyzed with open software ImageJ.

### Preparation of R6G-ZnONPs

R6G-ZnONP synthesis protocol was modified from previous papers and performed by the following steps [33, 70]. First, 1 ml of 1000 μg/ml ZnONPs was mixed with 5 μl of 1 mM rhodamine 6G (R6G, Sigma, #252,433). The mixture was sonicated for 30 min to conjugate ZnONPs and R6G. After sonication, the precipitate was collected by centrifugation at 13,500 rpm for 30 min. Then, the R6G-ZnONP solution was prepared by resuspending the pellet in distilled water. The chemical bonding and surface chemistry of R6G-ZnONPs were examined by using XPS. The XPS spectra of ZnONPs and R6G-ZnONPs were recorded by using an X-ray photoelectron spectrometer (PHI VersaProbe 4, Physical Electronics, Inc. USA) with an aluminum mono Kα source. To obtain information on each element, a high-resolution survey (pass energy 117.40 eV) was performed at spectral regions relating to zinc, carbon, and oxygen.

### Nanoparticle distribution in skin

Fluorescence microscopy was applied to visualize process of R6G-ZnONPs penetrate in skin. Mice were treated with the same process of in vivo study. After the exposure process, mice were sacrificed, the skin samples were fixed and dehydrated with 30% sucrose solution. The skin samples were embedded and freeze in OCT gel (Optimal cutting temperature gel) for the cryosection. The image of skin section was obtained with fluorescence microscope (Nikon H600L, Japan) and analyzed using the Nikon microscope software NIS-Elements.

In addition to detecting R6G-labeled ZnONPs in skin samples, direct detection of ZnONPs in skin samples was also performed by using multiphoton microscopy. According to a previous study, ZnONPs exhibit various fluorescence emissions due to their band structure (2.38–3.54 eV), and ZnONPs can be identified by green fluorescence emission [71]. Multiphoton microscopy was performed using a homemade system (Core Facility Center, National Cheng Kung University, Taiwan) that included a Chameleon Vision II titanium sapphire femtosecond laser source with a tuning range from 680 to 1600 nm (Chameleon, PA, USA). Fluorescence emission was detected using a Zeiss Plan-APOCHROMAT 20 × /0.8 objective lens with 420/10 nm (blue) and 531/40 nm (green) low-pass filters. Photon signals were collected by photomultiplier tubes (PMTs, H10721P-110, Hamamatsu, Japan). The image was mapped in a 200 × 200 μm (256 × 256 pixel) area. The image of the skin section was stitched together from four images by using ImageJ software.

### Acridine orange (AO) Staining

Acridine orange (AO) (Invitrogen, Carlsbad, CA, USA) was used to detect the acidic vesicular such as autophagolysosomes in cytosol. The detail staining protocol was followed by previous study [33]. Briefly, HaCaT cells were stained with AO solution (1 μg/ml) for 20 min at 37 °C after the treatment and washed once with PBS. After the incubation, cells were trypsinized and resuspended in PBS. The fluorescence was detected by using FACS Calibur flow cytometry (BD Biosciences, USA). Ten thousand cells in each sample. The raw data was analyzed by FlowJo 7.6.1 software.

### Immunofluorescence staining for detecting autophagic flux blockage

Cells were seeded on black clear bottom 96-well plate. After treatment, the cells were fixed with 4% paraformaldehyde and stained for nuclei (DAPI), autophagosomes (LC3) and lysosomes (LAMP-1), as described under “Immunofluorescence Staining and High‐Throughput Screen” (Supplementary information). The plates were assayed using ImageXpress® Micro Confocal High-Content Imaging System (Molecular Devices, San Jose, CA, USA). LC3 immunostaining was captured using a FITC filter and LAMP1 immunostaining using a TRITC filter. Images were subjected to segmentation and quantification using the custom module editor in MetaXpress analysis software.

### Exosome isolation

The exosome isolation protocol was followed by previous study [33]. THP-1 cells and HaCaT cells were cultured in Ultra CULTURE™ Serum-free Cell Culture Medium (Lonza, #12-725F) to avoid contamination of EVs from fetal bovine serum. After exposure, the culture medium was collected and exosomes were isolated via differential centrifugation. Briefly, the medium was centrifuged for 10 min at 3000×*g* to eliminate cell debris, then the supernatants was centrifuged for 30 min at 10,000×*g* to eliminate microvesicles. The exosomes were collected by using ultracentrifuge for 120 min at 100,000×*g*. (Optima L-100XP Ultracentrifuge (Beckman), SW28 rotor (Beckman)), the exosome pellets were resuspended in 0.2 μm filtered particle free PBS.

### NanoSight detection

Exosome samples were measured by using nanoparticle tracking analysis (NTA) (NanoSight, Amesbury, UK) to obtain the concentration and size distribution of exosomes [72]. Samples were diluted with 1000 μl PBS. Three hundred μl sample was acquired into the chamber of NTA unit (NanoSight, Amesbury, UK). The image data was analyzed with NTA 2.3 software (NanoSight, Amesbury, UK).

### Transwell experiment

To analysis exosome interaction between cells, a coculture system was used to mimic the cellular environment, the detailed protocol was described previously [73]. 4 × 10^4^ HaCaT cells were first cultured in the upper chamber of the 0.4 μm pores Transwell (Corning #3413). Then cells membrane was labeled with DioC18 dye (5 μg/ml; Invitrogen, D275) at 37 °C, 30 min. After staining, the cells were washed three times with PBS to remove excess dye. After the indication treatment of HaCaT cells. The HaCaT cells were cocultured with DioC18 unstained THP-1 cells (4 × 10^5^ cells/well, 100 ng/ml PMA differentiated) in two separate compartments of the system. DioC18-labeled exosomes from upper compartment cells were uptaken by the THP-1 cells in the lower compartment, the exosomes uptake rate was observed by fluorescence microscopy and quantified by flow cytometry analysis.

### Flow cytometry detection of THP-1 derived M1/M2 Macrophages

THP-1 cells were first differentiated with 100 ng/ml PMA (phorbol-12-myristate-13-acetate). After incubation with or without 100 ng/ml LPS and exosome for 24 h, cells were trypsinized and resuspended in PBS. Cells were centrifuged 5 min at 200×*g*, 4 °C. The pellet was resuspended in 100 μl PBS with 20 μl of primary APC-anti-CD206 (BD Biosciences, #550,889) and 20 μl of PE-anti-CD86 (BD Biosciences, #560,957) antibody and incubated for 30 min at 37 °C. Cells were also incubated with the control isotype corresponding to each primary antibody (PE Rat IgG2a, κ Isotype Control, #553,930) (Alexa Fluor® 647 Rat IgG2a, κ Isotype Control, #557,690). After incubation, cells were washed with PBS and recollected by centrifugation at 200×*g*, 4 °C for 5 min. Three washes with PBS were next performed. Cells were analyzed by flow cytometry with CytoFLEX Flow Cytometer (Beckman Coulter, USA).

### Immunofluorescence staining for paraffin embedding of skin tissue

The paraffin-embedded skin samples were first deparaffinization. Then the samples were recovered antigenicity by using the Tris–EDTA buffer and heated to 100 °C for 20–40 min. To block the autofluorescence, slides were incubated with 0.1% Sudan Black in 70% ethanol solution for 10 min at room temperature. The samples were stained with the indicated primary antibody against CD86 (Novus, NBP2-25,208) (1:200) and CD206 (abcam, ab64693) (1:200) for 1 h at 37 °C and incubated with Alexa Fluor 488- (abcam, ab150077) or Alexa Fluor 594- (abcam, ab150116) conjugated secondary antibody (1:200) for 1 h at 37 °C. The nuclei were stained by DAPI staining. Images were obtained with fluorescence microscope (Nikon H600L, Japan) and analyzed using the Nikon microscope software NIS-Elements.

### Immunohistochemistry staining for cryopreservation of skin tissue

The OCT compound-embedded skin samples were cut into cryostat sections at 5–10 µm and mounted on gelatin-coated histological slides. Then the samples were rehydrated, treated with 3% hydrogen peroxide for 5 min, and blocked the section with 1–3 drops of serum blocking reagent (Millipore, DAB500) for 1 h at room temperature. The tissue sections were incubated with primary antibodies against IL-1β (16,806–1-AP, Proteintech Group, Chicago, IL, USA) at 4 °C overnight. After washing, added aliquots of biotinylated secondary antibodies. The reaction was visualized by streptavidin HRP and DAB chromogen reagent. Then, the slides were stained with hematoxylin and were observed under a light microscope.

### Real-time quantitative polymerase chain reaction (qPCR)

THP-1 cells were exposed to 10 or 12.5 μg/ml ZnO NPs. After 18 h exposure, the RNA was isolated using TOOLSharp RNA Extractor (TTD-NRNA200, BIOTOOLS Co., Ltd., Taipei, Taiwan). RNA concentrations and 260/280 nm ratios were measured by NanoDrop 2000 (Thermo Scientific™, Waltham, MA, USA). Reverse transcription (RT) was performed using TOOLSQuant II Fast RT Kit (KRT-BA06-2, BIOTOOLS Co., Ltd., Taipei, Taiwan). Finally, a mixture of the synthesized cDNA, primers and TOOLS 2 × SYBR qPCR Mix (FPT-BB05, BIOTOOLS Co., Ltd., Taipei, Taiwan) was carried out in a StepOnePlus™ Real-Time PCR System (Applied Biosystems, Foster, CA, USA) using SYBR Green Master Mix (Thermo Fisher Scientific). The average of the ^ΔΔ^C(t) of triplicate samples was calculated using β-actin as a housekeeping gene. The sequences of forward primers and reverse primers were summarized as described under Supplementary information.

### Western blot analysis

Cell or tissue lysates were extracted in lysis buffer. The protein concentration was determined by BCA (bicinchoninic acid) assay. About 20 μg of protein extract was separated on 6–15% SDS–polyacrylamide gels and transferred to polyvinylidene difluoride membranes (Merck Millipore, Darmstadt, Germany). After 1 h blocking procedure, the target proteins were probed by corresponding primary antibodies in 1:1000 dilution. The information and resources of antibodies are provided in supplementary information. The membrane was washed with 1 × TBST after primary antibody hybridization, target proteins were reacted with HRP-conjugated anti-mouse or anti-rabbit secondary antibodies in 1:10,000 dilution. The immunoreactive proteins were visualized with chemiluminescence HRP substrate (Merck, USA) and filmed with BioMax LightFilm (Eastman Kodak Company, New Heaven, CT, USA). The band intensities were quantified by ImageJ software.

### Immunoprecipitation

After treatment with ZnONPs for indicated time points, THP-1-derived macrophage were lysed and the soluble fraction was incubated overnight at 4 °C with an antibody to anti-ASC (ABclonal Inc., Boston, MA, USA) and anti-NLRP3 (Cell Signaling, Beverly, MA, USA); then, A/G agarose beads (Merck Millipore, MA, USA) were mixed with the solution for further incubation for 1 h at 4 °C. The beads/pellets were washed three times with the protein extraction buffer, boiled in SDS sample buffer for 5 min, and centrifuged. The supernatant was subjected to western blot analysis using the indicated antibodies performed as described above.

### Antibodies

The antibodies for anti-NLRP3 (#15,101), ASC (#67,824), LC3-II (#4108), flotillin-1 (#3253), HSP70 (#4872), Cathepsin B (#31,718), p-p65 (#3033), p65 (#8242) and GAPDH (#2118) antibodies were purchased from Cell Signaling (Beverly, MA, USA); anti-caspase-1/p20 (22,915-1-AP) antibodies were purchased from Proteintech (Rosemont, IL 60018, USA); anti-LAMP-1 (NB120-19294), CD63 (NB100-77913) antibodies were purchased from Novus (Centennial CO 80112, USA); anti-TSG-101 (ab125011), p62 (ab109012), TNF-α (ab183218) and IL-1β (ab254360) antibodies were purchased from Abcam Inc. (Cambridge, MA, USA). anti-COX IV (tcna10591) was purchased from Taiclone Biotech Corp. (Taipei, Taiwan). The CD68 antibody (GTX41864) was purchased from GeneTex (Hsinchu City, Taiwan). The anti-iNOS (A0312) and anti-ARG1 (A4923) were purchased from ABclonal technology (Wobure. MA, USA). The HRP-conjugated anti-mouse (AB_10015289) and anti-rabbit (AB_2313567) secondary antibodies were purchased from Jackson ImmunoResearch Lab (West Grove, PA, USA).

### Detecting cytosolic release of lysosomal proteases

The HaCaT cells were exposed to ZnONPs (12.5 µg/ml) or UVB (68 mJ/cm^2^) for indicated times. To separate the cytosolic fraction (lysosome-free) from HaCaT cells, we used proper volume cold hypotonic buffer (10 mM HEPES − NaOH (pH 7.9), containing 10 mM KCl, 1.5 mM MgCl_2_, 1 mM DTT, 1 × protease inhibitor, 1 × phosphatase inhibitor) to dissolve the HaCaT cell pellets. The cell suspensions were incubated on ice for 30 min, mixing for 10 s every 5 min. After centrifugation at 400×*g* for 10 min at 4 °C, the supernatants were collected as cytosolic fractions. To extract the membrane fractions, (containing lysosomes and mitochondria) the cold MSC-RIPA buffer (20 mM Tris − HCl (pH 7.4) containing 100 mM NaCl, 6.05 M EG, 0.5% SDC, 0.05% SLS, 1 × protease inhibitor, 1 × phosphatase inhibitor) was added to the pellets and 4 °C incubation for 30 min. After centrifugation at 400×*g* for 10 min at 4 °C, the supernatants were collected as membrane fractions. Then, the samples were subjected to western blot analysis using the Cathepsin B (1:1000), COX IV (1:5000), and LAMP-1 (1:1000) antibodies performed as described above.

### Statistical analysis

SigmaPlot 10.0 (Systat Software, Inc., USA) was used to analyze the data and the results were presented as mean ± standard deviations (SD). The differences between two groups or multiple groups were evaluated using a two-sample t-test or one-way analysis of variance with a post hoc Dunnett’s multiple comparison test, respectively. *P* value < 0.05 was considered statistically significant difference.

### Supplementary Information

**Additional file 1**. Supplementary information of experimental section and figures.

**Additional file 2**. Raw images of western blot experiment.

## Data Availability

All data generated or analyzed during this study are included in this published article (and its supplementary information file).
