# Supplementary material for: Zinc oxide nanoparticles exacerbate skin epithelial cell damage by upregulating pro-inflammatory cytokines and exosome secretion in M1 macrophages following UVB irradiation-induced skin injury
Source: Part Fibre Toxicol. 2024 Feb 28;21:9. doi: 10.1186/s12989-024-00571-z (PMC10900617; doi:10.1186/s12989-024-00571-z)
Supplement: Supplementary file 1 — Additional file 1. Supplementary information of experimental section and figures. [file 12989_2024_571_MOESM1_ESM.docx]

**Zinc oxide nanoparticles exacerbate skin epithelial cell damage by upregulating pro-inflammatory cytokines and exosome secretion in M1 macrophages following UVB irradiation-induced skin injury**

Bour-Jr Wang, Yu-Ying Chen, Hui-Hsuan Chang, Rong-Jane Chen, Ying-Jan Wang, and Yu-Hsuan Lee

**Supplementary Information**

**Materials and Methods**

**Mice**

10-12 weeks Female SKH:HR-1 mice were used and acclimatized for a week before the experiment started. The drinking water and standard rodent laboratory chow (No. 5001; Laboratory Rodent Diet, Texas, USA) were free to access. Mice were housed five per cage at 12-hours light/dark cycle and kept at 24±2°C. Animal experiments were approved and supervised by the Institutional Animal Care and Use Committee of National Cheng Kung University, Taiwan (Approval No.:108225).

**Cell Culture**

The source of cells is provided in the Supplementary Information. The HaCaT cells were cultured in DMEM (Gibco, NY, USA) supplemented with 10% fetal bovine serum (Gibco, NY, USA), 5% CO2 at 37 °C In in vitro study, HaCaT cells received a single dose of UVB radiation (40, 50, 68 mJ/cm^2^) by using UVB Crosslinker (UVP CL-1000). After the UVB exposure, cells were treated with ZnO NPs in different dose (5, 10, 12.5 or 15 μg/ml) for 24 hours.

The THP-1 cells were cultured in RPMI 1640 (Gibco, NY, USA) supplemented with 10% fetal bovine serum (Gibco, NY, USA), 5% CO2 at 37 °C. THP-1 cells were treated with ZnO NPs under the indicated dose (1-50 μg/ml) for 24 and 48 hours.

**Cell Viability Assay**

HaCaT cells were seeded in 12 wells (1×10^5^/well) and treated with different concentrations of ZnO NPs (5, 10, 12.5, or 15 μg/ml) and UVB radiation (40, 50 or 68 mJ/cm^2^) for 24 h. THP-1 cells were seeded and differentiated with 100 ng/ml PMA in12 wells (1×10^5^/well) for 24 h and treated with different concentrations of ZnO NPs (1-45 μg/ml) for a further 24 and 48 hours. After exposure, the cells were trypsinized and collected for mixing with 0.4% trypan blue solution. The trypan blue/cell 1:1 mixture was applied to the viable/death cell counting

**ROS Analysis:**

H_2_DCF-DA staining was used to detect the cellular total ROS generation (Sigma-Aldrich, #D6883). Cells were first treated with 50 μM H_2_DCF-DA in medium, incubated at 37°C for 30 min then washed with PBS. Cells were trypsinized and resuspended in PBS. The fluorescence was detected by using FACS Calibur flow cytometry (BD, San Jose, CA, USA). Ten thousand cells were collected in each sample. The raw data was analyzed by FlowJo 7.6.1 software.

**Lysosensor Staining and Confocal Microscopy Analysis**

HaCaT cells and THP-1 cells were seeded on coverslip-bottom dishes. After treatment of R6G-ZnO NPs, the cells were treated with 50 nM Lysosensor (Invitrogen, L7535) and incubated at and washed with PBS. The nuclei were stained by DAPI. Images were obtained with an TCS SP8 confocal microscope (Leica Microsystems, Inc., Germany) and analyzed using the Zeiss confocal software ZEN 2010.

**Immunofluorescence Staining and High‐Throughput Screen**

HaCaT cells and THP-1 cells were seeded on black clear bottom 96-well plate. After ZnONPs alone or combined treatment, the cells were fixed with 4% paraformaldehyde for 10 min, washed 3 times with PBS and treated with 0.5% Triton X-100 (Sigma, T8787) to penetrate the cell membranes. The samples were stained with the indicated primary antibody against LC3 (Cell Signaling (Beverly, MA, USA)) (1:200) and LAMP-1 (Novus (Centennial CO 80112, USA)) (1:200) for 1 hour at 37°C and incubated with Alexa Fluor 488- or Alexa Fluor 594-conjugated secondary antibody (1:200) for 1 hour at 37°C. The nuclei were stained by DAPI staining. Images were obtained with ImageXpress® Micro Confocal High-Content Imaging System (Molecular Devices, San Jose, CA, USA) and analyzed using the MetaXpress software.

**Real-Time Quantitative Polymerase Chain Reaction (qPCR)**

The sequences of forward primers and reverse primers were summarized as follows:

| TLR-1 | Forward: 5’-TCCACGTTCCTAAAGACCTATCC-3’  Reverse: 5’-GGTTCACAGTAGGGTGGCAA-3’ |
| --- | --- |
| TLR-3 | Forward: 5’-TTGCCTTGTATCTACTTTTGGGG-3’  Reverse: 5’-TCAACACTGTTATGTTTGTGGGT-3’ |
| TLR-6 | Forward: 5’-CATGTTCCAAAAGACCTACCGC-3’  Reverse: 5’-ACTCACAATAGGATGGCAGGATA-3’ |
| TNF-α | Forward: 5’-AGCCCACGTCGTAGCAAACCACCAA-3’  Reverse: 5’-ACACCCATTCCCTTCACAGAGCAAT-3’ |
| IL-1β | Forward: 5’-CAGCTACGAATCTCCGACCAC-3’  Reverse: 5’-GGAAGGGAACCAGCATCTTC-3’ |
| β-actin | Forward 5’-AAGAGAGGCATCCTCACCCT-3’  Reverse 5’-TACATGGCTGGGGTGTTGAA-3’ |

**Supplementary Figures**

**

Figure S1. Fluorescence emission spectrum of ZnONPs.** ZnONPs were excited with 355 nm wavelength. The spectrum recorded in the emission range 400-850 nm with slit width fixed at 10 nm. The main emission fluorescence was around 500-600 nm (green-yellow color).

**
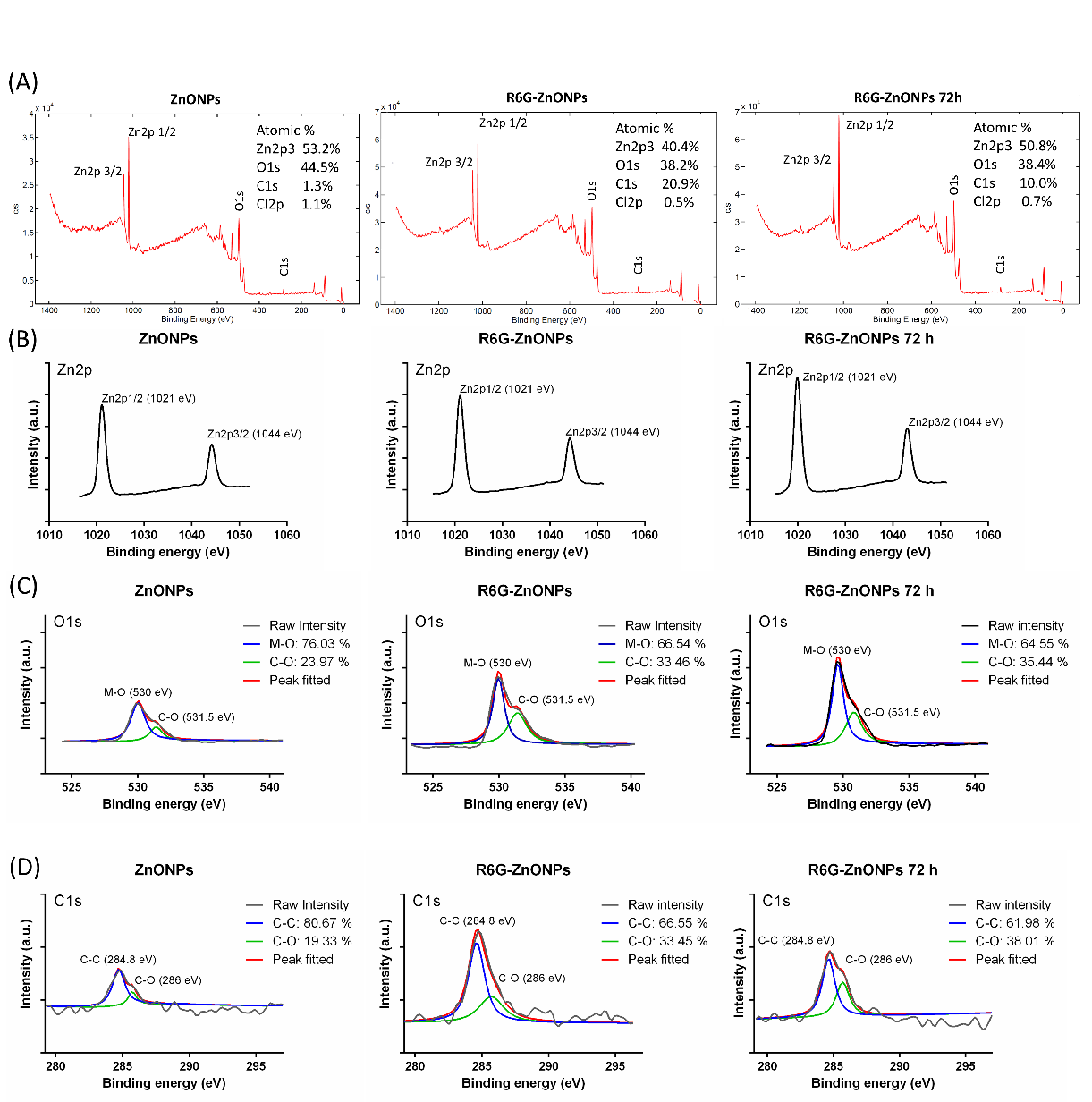
**

**Figure S2. XPS spectra of ZnONPs and R6G-ZnONPs.** The X-ray photoelectron spectroscopy (XPS) was used to study the surface chemistry of ZnONPs and the R6G-ZnONPs. (A) XPS survey spectrum of ZnONPs, R6G-ZnONPs, and R6G-ZnONPs 72h. (B) XPS core level scan of Zn2p_1/2_ and 2p_3/2_. The peak positions of Zn 2p1/2 (1021 eV) and Zn 2p3/2 (1044 eV) with binding energy difference in 23 eV was indicating as a characteristic value of ZnO. (C) O1s core level scan, existence of metal oxide (M-O) and C-O is shown through deconvoluted profiles. The O1s spectrum also showed increasing C-O group in R6G-ZnONPs (33.46 %) with decreasing metal oxide group (M-O, 66.54 %), compared to ZnONPs (C-O 23.97 %, M-O 76.03 %) (D) C1s core level scan, existence of C-C and C-O is shown through deconvoluted profiles. The peak (C-C) of C1s spectrum is located at 284.8 eV and the peak at 286 eV can be assigned to C-O functional group in R6G-ZnONPs, which accounted for 33.45 % compared to 19.33% in ZnONPs group.

**
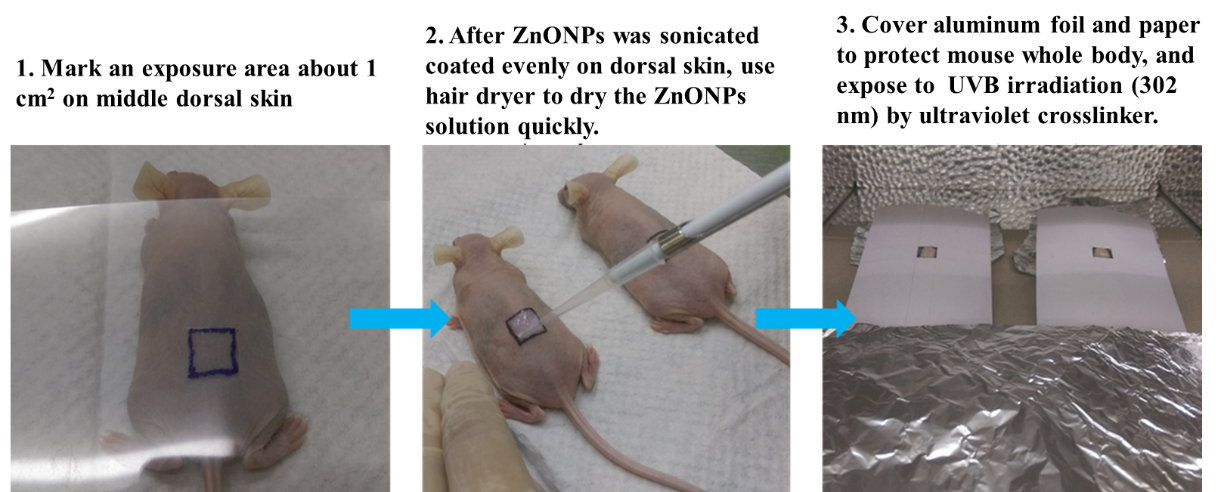
**

**Figure S3.** **Experimental design of applying ZnONPs to animal skin. Schematic diagram of operation.**


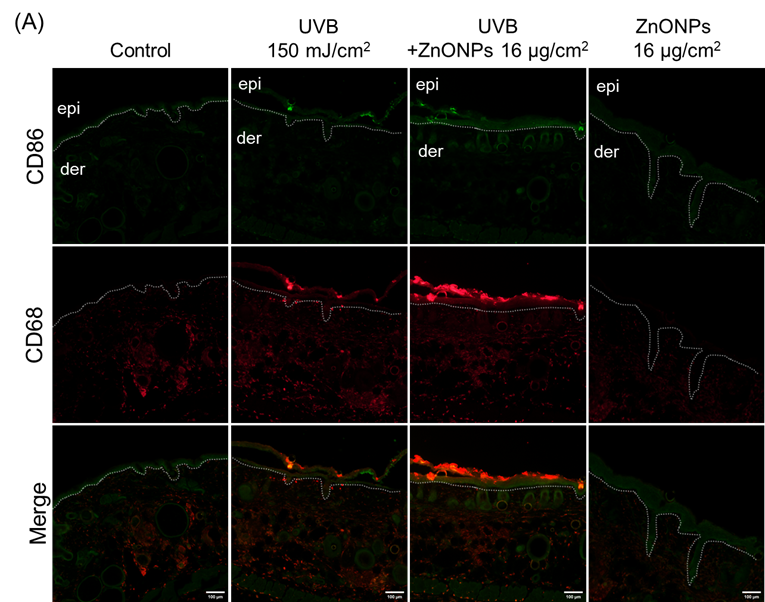


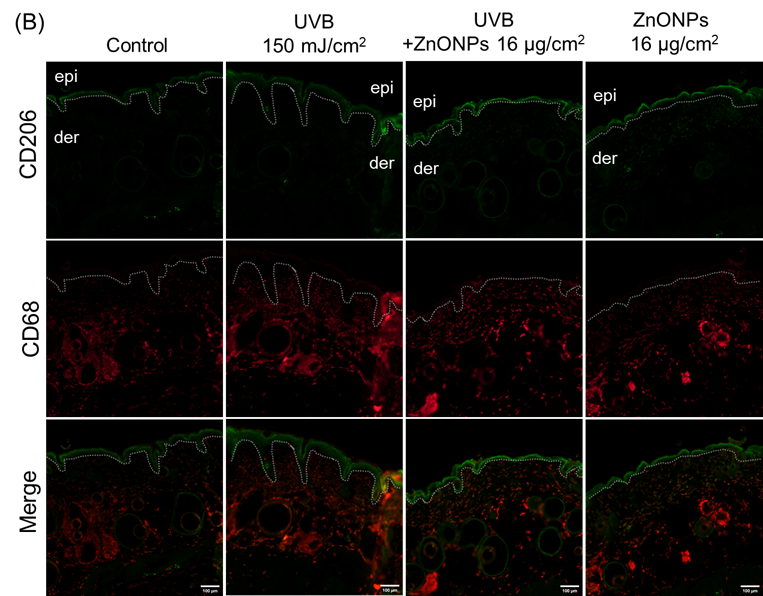


**Figure S4. Representative immunofluorescent staining of M1 (CD68^+^CD86^+^) and M2 (CD68^+^CD206^+^) macrophages in different groups.** Dotted line indicated the border between epidermis and dermis. Scale bars: 100 μm.


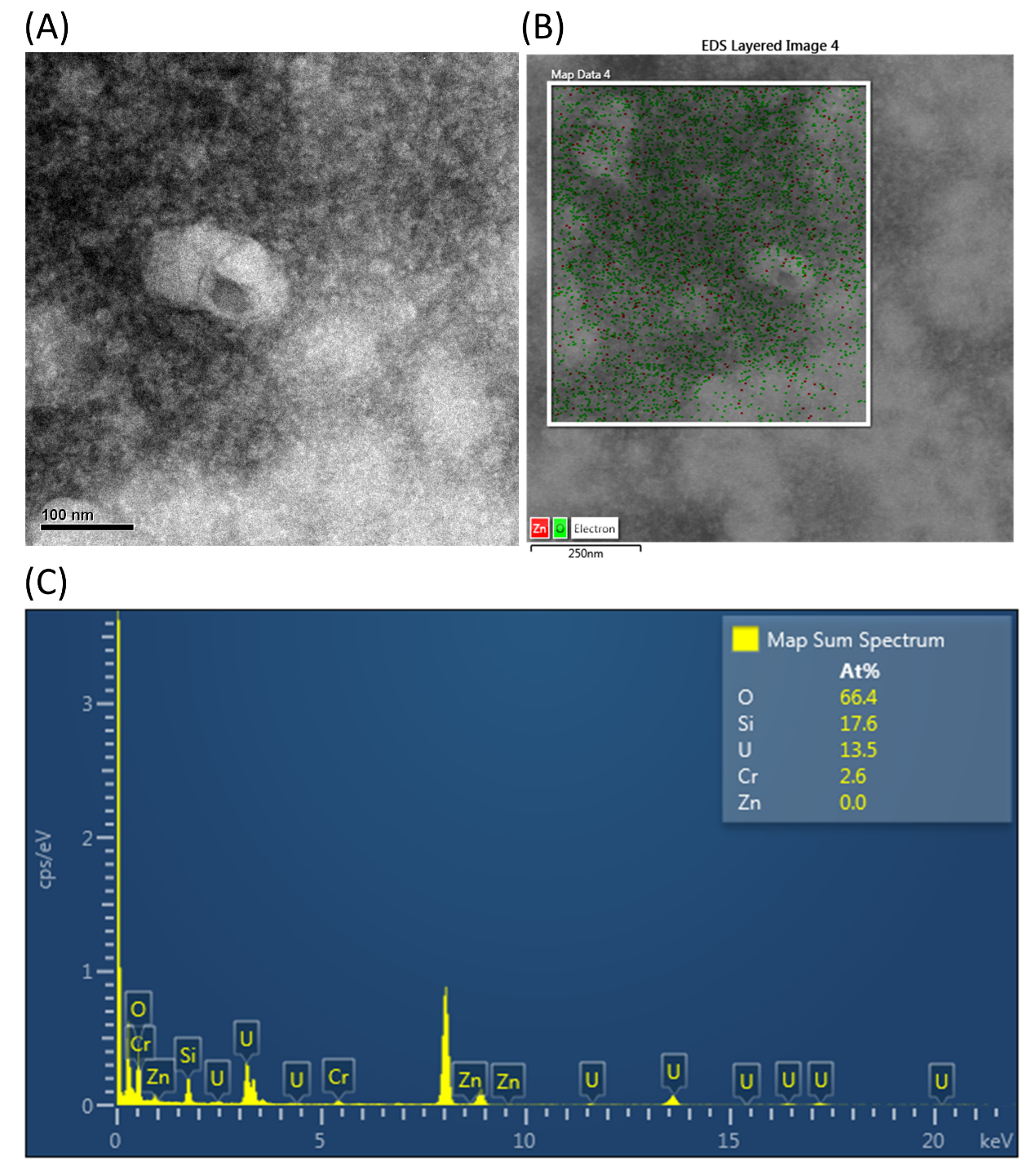


**Figure S5. Characterization of UVB + ZnONPs exposure HaCaT cells-derived exosomes.** (A) TEM micrograph of exosome stain with uranyl acetate (scale bar: 100 nm) and (B) related EDS element maps (scale bar: 200 nm) of zinc (Zn), oxygen (O) element and (C) related EDS spectrum.


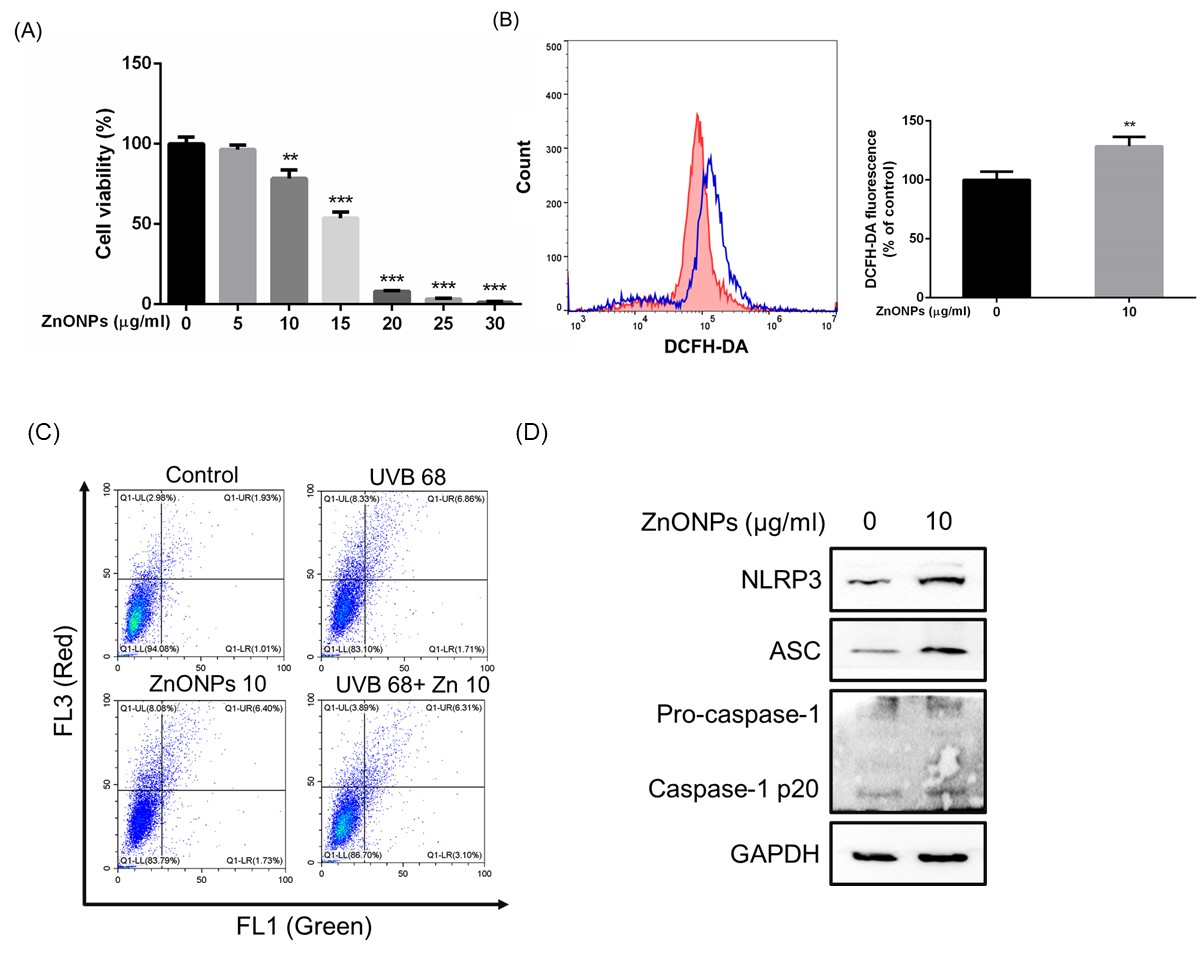


**Figure S6.** **Cell viability, ROS generation, autophagy induction, and NLRP3 inflammasome activation of ZnONPs reference material.** ZnONPs (product number 721077, Sigma-Aldrich) of <100 nm particle size (by dynamic light scattering, DLS) were used in this study to verify the general applicability of mechanism in this study. (A) HaCaT cell viability was examined after 24 hrs exposure. (**p < 0.01, ***p < 0.001 compared with control). (B) ROS generation of THP-1-derived macrophages after 0.5 hr exposure of 10 μg/ml ZnONPs. (C) Acridine orange staining of ZnONPs, UVB, and combine treatment of HaCaT cells at 24 hrs. (D) NLRP3, ASC, caspase-1 and caspase-1 p20 were analysed after ZnONPs exposure at 24 hrs, GAPDH represented as loading control.


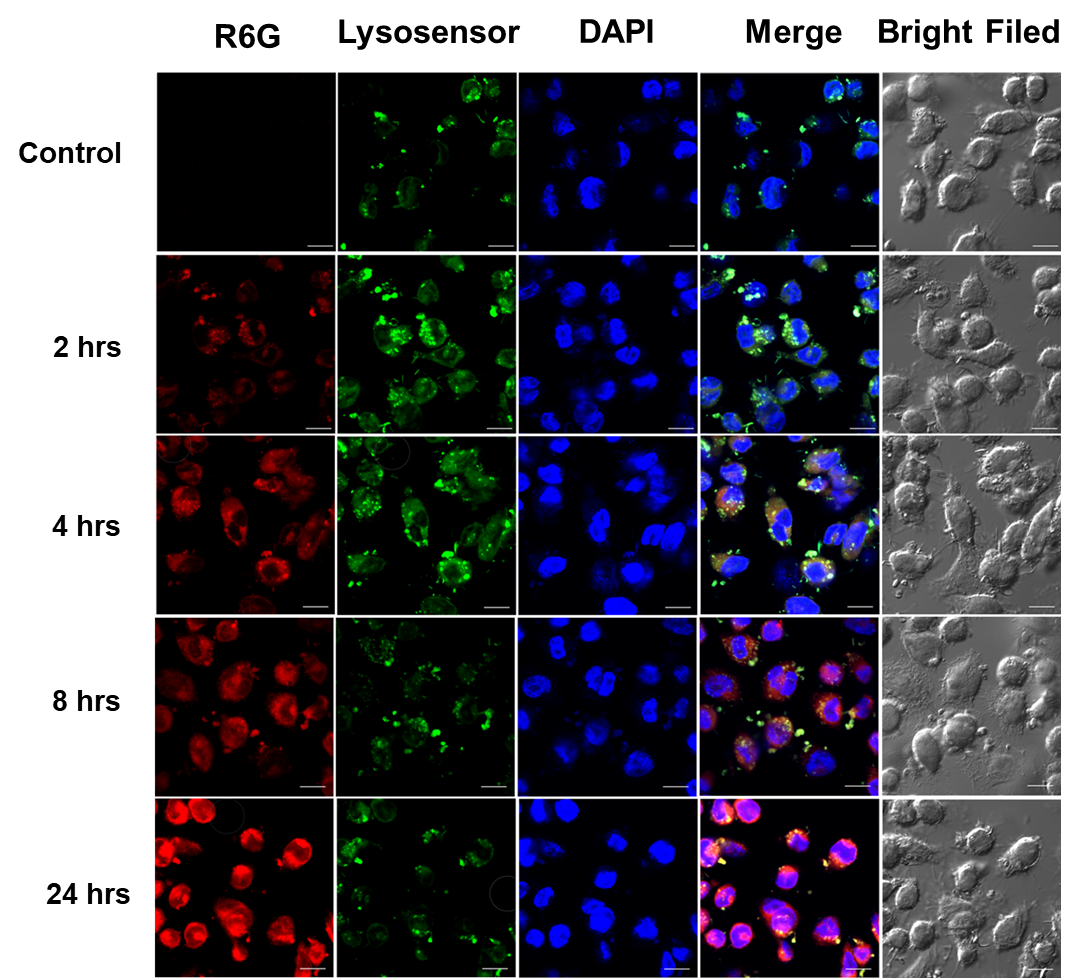


**Figure** **S7. ZnONPs disrupted the function of lysosome.** THP-1 cells were differentiated with PMA for 24 hrs and then exposed to 10 μg/ml ZnONPs for various times. Fluorescence analysis of R6G-labeled ZnONPs and lysosomal activity in the cells under a time-course of ZnONPs exposure. Lysosomal activity was detected by Lysosensor DND-189 staining. Scale bars: 50 μm. Cellular uptake of ZnONPs was detected by measuring the red fluorescence of R6G-labeled ZnONPs. DAPI staining represented the nuclear DNA of cells.
