# Supplementary material for: Zinc oxide nanoparticles exacerbate skin epithelial cell damage by upregulating pro-inflammatory cytokines and exosome secretion in M1 macrophages following UVB irradiation-induced skin injury
Source: Part Fibre Toxicol. 2024 Feb 28;21:9. doi: 10.1186/s12989-024-00571-z (PMC10900617; doi:10.1186/s12989-024-00571-z)
Supplement: Supplementary file 2 — Additional file 2. Raw images of western blot experiment. [file 12989_2024_571_MOESM2_ESM.pdf]

Figure 3

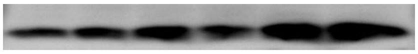

Fig. 3D_ LC3

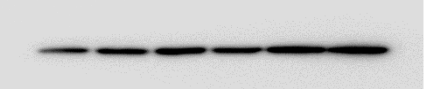

Fig. 3D_ GAPDH

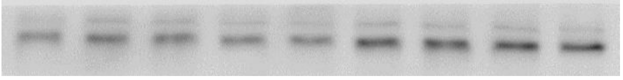

Fig. 3G_ cytosolic_Cathepsin B

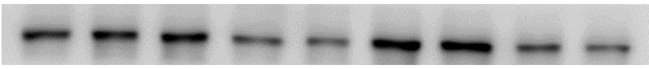

Fig. 3G_ membrane_Cathepsin B

Fig. 3G_ cytosolic_LAMP-1

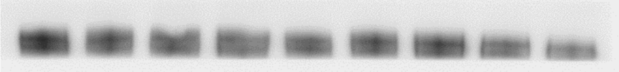

Fig. 3G_ membrane _LAMP-1

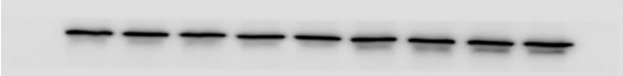

Fig. 3G_ cytosolic_GAPDH

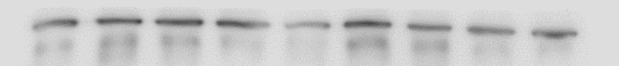

Fig. 3G_ membrane _GAPDH

Fig. 3G_ cytosolic_Cox IV

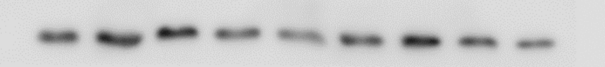

Fig. 3G_ membrane _Cox IV

Figure 4

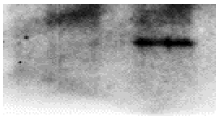

Fig . 4A_CD63

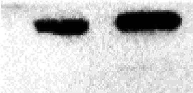

Fig. 4A_TSG101

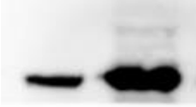

Fig. 4A_Flotillin-1

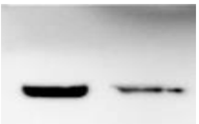

Fig. 4A_HSP70

Figure 5

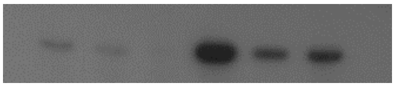

Fig . 5D_p-p65

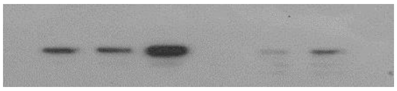

Fig . 5D_p65

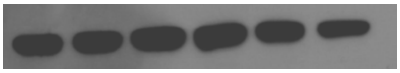

Fig . 5D_GAPDH

Figure 6

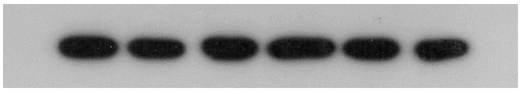

Fig. 6A_ GAPDH

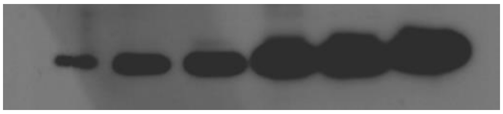

Fig. 6A_ LC3

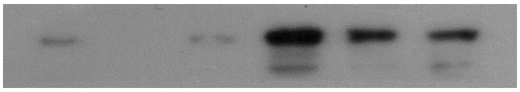

Fig. 6D_ NLRP3

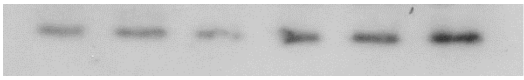

Fig. 6D_ ASC

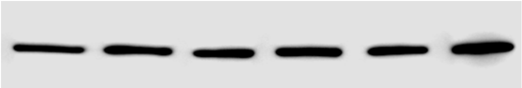

Fig . 6D_GAPDH

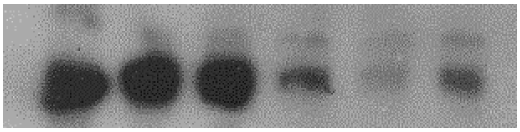

Fig. 6D_ Pro-caspase 1

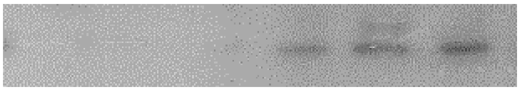

Fig. 6D_ Caspase 1 p20

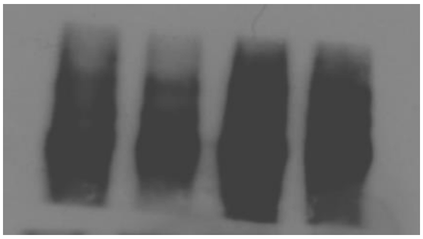

Fig.6E_ IP: ASC
IB: NLRP3

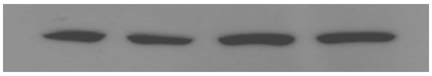

Fig.6E_ NLRP3

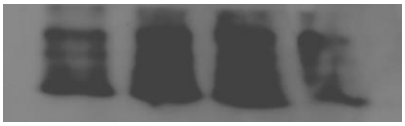

Fig.6E_ IP: NLRP3
IB: ASC

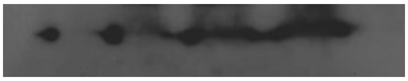

Fig.6E_ ASC

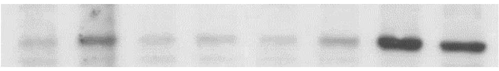

Fig.6G_ NLRP3

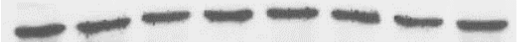

Fig.6G_ GAPDH

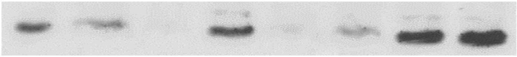

Fig.6G_ LC3

Figure 7

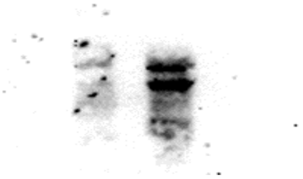

Fig. 7A_ CD63

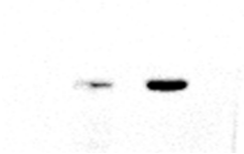

Fig. 7A_ TSG101

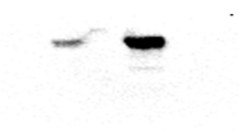

Fig. 7A_ Flotilin-1

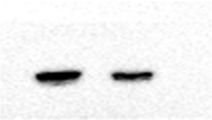

Fig. 7A_ HSP70

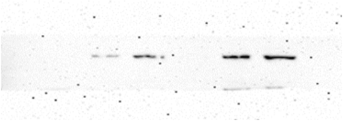

Fig. 7C_ NLRP3

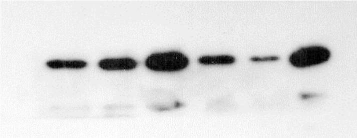

Fig. 7C_ ASC

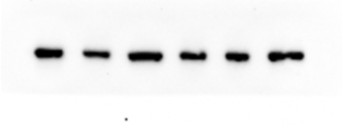

Fig. 7C_ Flotillin-1

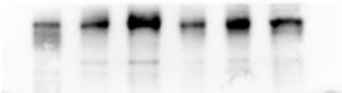

Fig. 7C_ Caspase-1

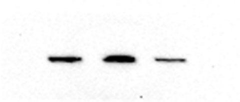

Fig. 7D_ NLRP3

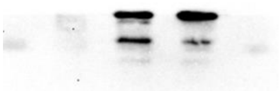

Fig. 7D_ IL-1β

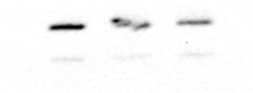

Fig. 7D_ ASC

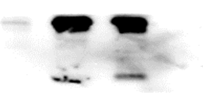

Fig. 7D_ TNF-α

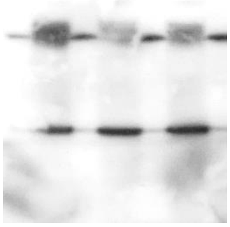

Fig. 7D_ Caspase-1
& caspase-1 p20

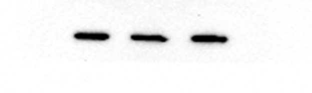

Fig. 7D_ GAPDH
